# *Fusarium Oxysporum* Volatiles Enhance Plant Growth Via Affecting Auxin Transport and Signaling

**DOI:** 10.3389/fmicb.2015.01248

**Published:** 2015-11-10

**Authors:** Vasileios Bitas, Nathaniel McCartney, Ningxiao Li, Jill Demers, Jung-Eun Kim, Hye-Seon Kim, Kathleen M. Brown, Seogchan Kang

**Affiliations:** ^1^Department of Plant Pathology and Environmental Microbiology, The Pennsylvania State University, University ParkPA, USA; ^2^Department of Entomology, The Pennsylvania State University, University ParkPA, USA; ^3^Center for Chemical Ecology, The Pennsylvania State UniversityUniversity Park, PA, USA; ^4^Intercollege Graduate Degree Program in Plant Biology, The Pennsylvania State UniversityUniversity Park, PA, USA; ^5^Department of Plant Science, The Pennsylvania State UniversityUniversity Park, PA, USA

**Keywords:** auxin signaling, chemical effector, fungal ecology, plant growth and development, volatile organic compounds

## Abstract

Volatile organic compounds (VOCs) have well-documented roles in plant-plant communication and directing animal behavior. In this study, we examine the less understood roles of VOCs in plant-fungal relationships. Phylogenetically and ecologically diverse strains of *Fusarium oxysporum*, a fungal species complex that often resides in the rhizosphere of assorted plants, produce volatile compounds that augment shoot and root growth of *Arabidopsis thaliana* and tobacco. Growth responses of *A. thaliana* hormone signaling mutants and expression patterns of a GUS reporter gene under the auxin-responsive DR5 promoter supported the involvement of auxin signaling in *F. oxysporum* volatile-mediated growth enhancement. In addition, 1-naphthylthalamic acid, an inhibitor of auxin efflux, negated *F. oxysporum* volatile-mediated growth enhancement in both plants. Comparison of the profiles of volatile compounds produced by *F. oxysporum* strains that differentially affected plant growth suggests that the relative compositions of both growth inhibitory and stimulatory compounds may determine the degree of plant growth enhancement. Volatile-mediated signaling between fungi and plants may represent a potentially conserved, yet mostly overlooked, mechanism underpinning plant-fungus interactions and fungal niche adaption.

## Introduction

Organisms in multiple kingdoms use volatile metabolites as semio-chemicals for intra- and inter-species communication and manipulation. Such function of various volatiles produced by animals and plants has been well-documented (Baldwin et al., 2002; Herrmann, 2010). Although available data on microbial semio-volatiles, especially those produced by fungi, is rather limited, they also seem to participate in modulating sexual reproduction, controlling physiology and growth within and between species, antagonizing other organisms, and coordinating symbiosis (Tsavkelova et al., 2006; Young, 2009; Bennett et al., 2012; Morath et al., 2012; Bitas et al., 2013). Volatile compounds produced by certain plant growth promoting rhizobacteria enhanced plant growth, induced systemic resistance against pathogens and/or increased abiotic stress tolerance (Ryu et al., 2003, 2004; Han et al., 2006; Zhang et al., 2007; Kwon et al., 2010). In contrast, bacteria, such as strains of *Serratia* spp., *Pseudomonas* spp. and *Stenotrophomonas* spp., produce volatiles that inhibit the growth of *Arabidopsis thaliana* (Vespermann et al., 2007; Kai and Piechulla, 2009). Volatiles from *Trichoderma viride*, a biocontrol fungus, augmented *A. thaliana* growth (Hung et al., 2013), and volatiles produced by *Phoma* sp. enhanced tobacco growth (Naznin et al., 2013). Similarly, a biocontrol strain of *Fusarium oxysporum* that carries ectosymbiotic bacteria produced β-caryophyllene, a volatile sesquiterpene that appeared to enhance lettuce growth (Minerdi et al., 2011). However, when its symbionts were removed, the strain became pathogenic, ceased producing β-caryophyllene, and failed to enhance plant growth. Volatiles produced by ectomycorrhizal truffles (*Tuber* spp.) inhibited *A. thaliana* root growth and development (Splivallo et al., 2007).

In this study, we characterized how volatile compounds produced by diverse isolates of *F. oxysporum* affect plant growth using *A. thaliana* and tobacco. Collectively, members of this cosmopolitan soilborne fungal species complex cause diseases in >100 plant species (Kang et al., 2014) by invading through the roots and subsequently blocking water and mineral movement through the xylem (Czymmek et al., 2007; Michielse and Rep, 2009; Rispail and Di Pietro, 2009). Pathogenic strains typically exhibit narrow host specificity, causing disease only in a single, or closely related plant species (Kang et al., 2014); such host-specialized groups of pathogen isolates are classified as formae speciales. They also asymptomatically colonize a wide spectrum of plants (Michielse and Rep, 2009). Strains that belong to diverse *formae speciales* were screened to assess how their volatiles affect plant growth. We demonstrate here that volatiles from *F. oxysporum* can stimulate growth of plants and that auxin participates in the responses to these volatiles. How such volatiles contribute to *F. oxysporum* rhizosphere competency and pathogenesis is also discussed.

## Materials and methods

### Fungal cultures and plant materials

Strains used in this study and their origins are listed in Table 1. They were stored as conidial suspension in 20% glycerol at −80°C and were revitalized by inoculating on half-strength Potato Dextrose Agar (PDA) at room temperature. Seeds of *A. thaliana* ecotypes were acquired from Lehle Seed Co. (Round Rock, TX, USA). The mutants of Col-0 used in this study, obtained from the Arabidopsis Biological Resource Center at Ohio State University, included *etr1* (Bleecker et al., 1988; Chang et al., 1993), *ga3ox1* and *aux1* (Marchant et al., 1999), *axr5* and *tir1* (Yang et al., 2004), and *eir1* (Luschnig et al., 1998). Transgenic Col-0 containing *DR5::GUS* (Jefferson et al., 1987) was provided by Darrell Desveaux at University of Toronto. Sara May at Penn State provided seeds of *Nicotiana tabacum* variety Samsun.

**Table 1:** ***F. oxysporum* strains screened in this study**.

**Accession #^a^**	**f. sp.^b^**	**Geographical origin**
7802	*ciceris*	Spain
8012	*ciceris*	Spain
9605	*ciceris*	Tunisia
W6-1	*ciceris*	California, USA
NRRL 38272	*conglutinans*	Australia
NRRL 38340	*conglutinans*	Unknown
FRC O-210	*conglutinans*	New York, USA
NRRL 38341	*conglutinans*	Unknown
NRRL 38342	*conglutinans*	Unknown
NRRL 38491	*conglutinans*	Unknown
FRC O-1115	*conglutinans*	California, USA
NRRL 36364	*conglutinans*	Unknown
NRRL 34936	*lycopersici*	Spain
NRRL 36467	*lycopersici*	Unknown
NRRL 38499	*lycopersici*	Unknown
NRRL 26383	*lycopersici*	Florida, USA
NRRL 38445	*lycopersici*	Unknown
NRRL 38550	*lycopersici*	Unknown
NRRL 36570	*radicis-lycopersici*	Unknown
NRRL 36572	*radicis-lycopersici*	Unknown
NRRL 26379	*radicis-lycopersici*	Florida, USA
NRRL 38343	*mathioli*	Unknown
NRRL 22545	*mathioli*	Germany
NRRL 22553	*raphani*	Germany
NRRL 38337	*raphani*	Unknown
NRRL 37616	*pisi*	Unknown
NRRL 37611	*pisi*	Australia
NRRL 36573	*phaseoli*	Unknown
NRRL 38282	*medicaginis*	N. Carolina, USA
NRRL 26411	*fabae*	Unknown
NRRL 36118	*cubense*	Unknown
NRRL 26029	*cubense*	Unknown
NRRL 36113	*cubense*	Unknown
NRRL 22519	*melonis*	Unknown
NRRL 36472	*melonis*	Unknown
NRRL 22557	*vasinfectum*	Unknown
NRRL 32599	*vasinfectum*	Australia
NRRL 36385	*cucurbitacearum*	Unknown
NRRL 36470	*dianthi*	Unknown
NRRL 26874	*spinaciae*	Unknown
NRRL 38507	*zingiberi*	Unknown
NRRL 38335	*tuberosi*	Korea
NRRL 38275	*caladium*	Florida, USA
NRRL 28973	*asparagi*	Korea
NRRL 26844	*lactucum*	Unknown
NRRL 39464	*carnation*	Korea

a *NRRL corresponds to ARS Culture Collection at the National Center for Agricultural Utilization Research and FRC indicates Fusarium Research Center at Penn State. The first four strains were provided by Dr. Maria Jimenez-Gasco at Penn State*.

b *Forma specialis or source of isolation*.

### I-plate assay for plant growth enhancement by fungal volatiles

Surface-sterilized seeds (soaked for 1 min in 95% ethanol, rinsed three times with sterile distilled water, soaked for 15 min in 5% sodium hypochlorite solution, and rinsed three times with sterile distilled water) were kept in sterile distilled water for 2 days at 4°C in the dark. Sterilized seeds were inoculated on half-strength Murashige and Skoog (MS) salts medium supplemented with 0.8% (w/v) agar and 2.5% (w/v) sucrose (Murashige and Skoog, 1962) in 110 × 110 mm square Petri dishes (VWR, Radnor, PA, USA). These plates were placed in a growth chamber (Conviron PGR15, Winnipeg, MB, Canada) set at 22°C, 12 h light (4500 lux, 60 μmol photons m^−2^ s^−1^) and 60% relative humidity.

**Figure 1:**
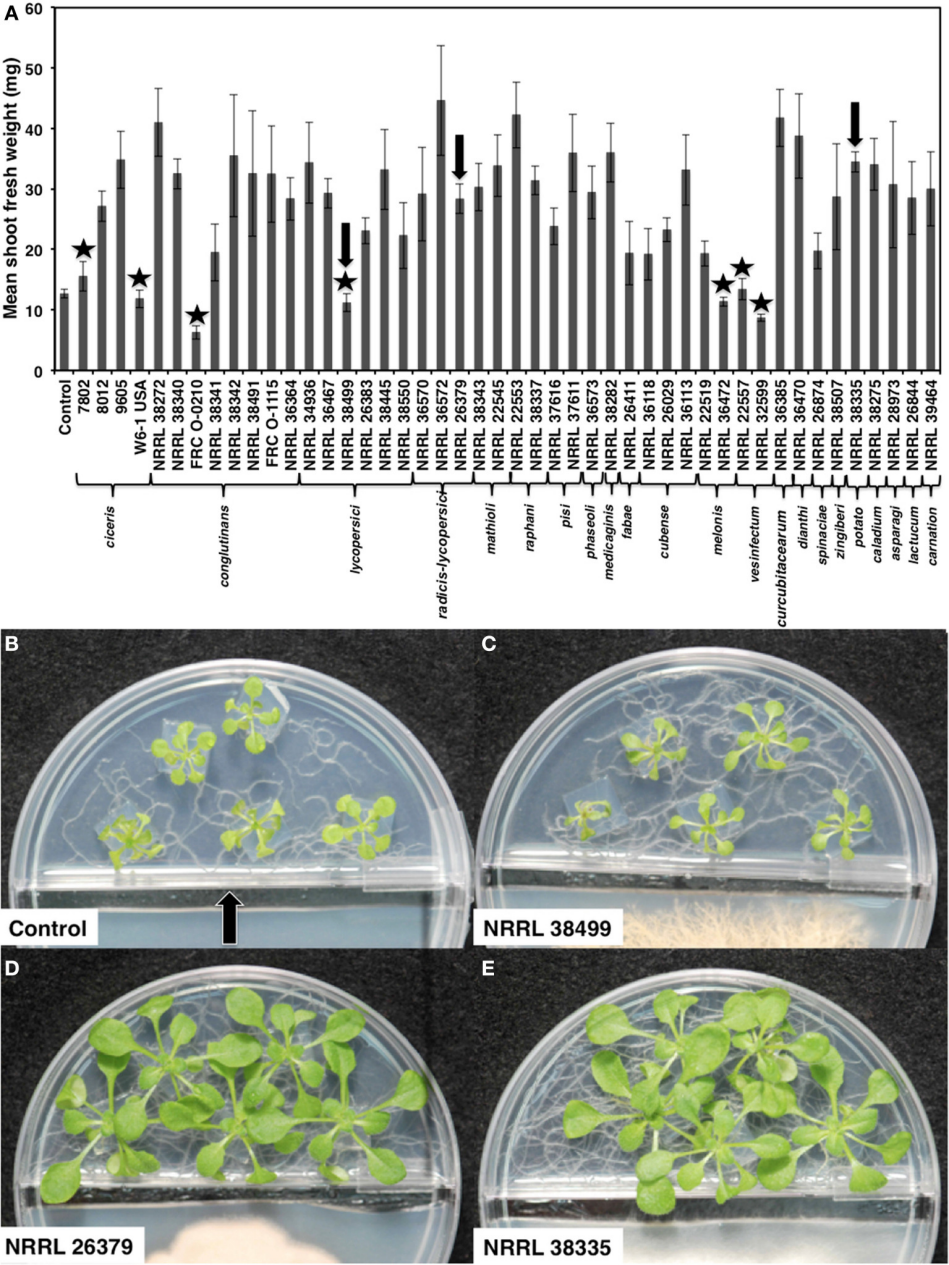
**Growth response of *A. thaliana* to *F. oxysporum* volatiles**. **(A)** Mean shoot fresh weights of ecotype Col-0 cocultivated with no fungus (control) and 46 *F. oxysporum* strains individually for 2 weeks are shown. Means and standard errors for nine biological replicates per treatment, with five seedlings per replicate, are shown. Isolates that did not cause statistically significant growth enhancement compared to control (Tukey's test, α < 0.05) are marked with star. Arrows denote the three strains used for subsequent analyses. Images of seedlings after cocultivation with these three strains are shown in **(B–E)**. The center partition, marked by an arrow in **(B)** prevents physical contact between two compartments. A strip of fungal culture medium along the center partition was removed at the time of fungal inoculation to prevent fungal overgrowth to the plant side.

One compartment of the I-plate contained half-strength PDA for culturing *F. oxysporum*, and the other had the MS medium described above for growing plants. One day before seedling transplantation, one plug of fungal culture (5 mm in diameter) was inoculated on the PDA side, and a 0.5 cm-wide strip of PDA along the center partition was removed (Figure 1) to prevent fungal overgrowth to the MS side. Five *A. thaliana* seedlings with similar size and growth stage were transplanted to the MS side by cutting and transferring 1 cm^2^ agar blocks containing one seedling each. The control treatment for all experiments consisted of PDA alone. Inoculated I-plates were sealed with Parafilm and placed in a growth chamber under the conditions described above.

### Measurement of changes caused by *F. oxysporum* volatiles

After cocultivation, each plant was removed, and its roots were detached from the shoot. After removing any excess moisture on the leaves using a paper tissue, each shoot was weighed immediately. Leaf area, root length and the lateral root density were measured using the image analysis software WinRHIZO (Regent Instruments Inc., Ch Ste-Foy, Quebec, Canada).

Chlorophyll content was measured spectrophotometically as previously described (Hiscox and Israelstam, 1979). After measuring shoot fresh weight, they were placed in a sterile 15 mL tube containing 5 mL dimethyl sulfoxide (DMSO). This tube was incubated at 65°C for 8 h and then stored in the dark at 0 to −4°C until absorbance measurement. Absorbance was measured at 645 and 663 nm, and the total chlorophyll content (mg per gram of shoots) was calculated using the following formula: (A_645_ × 0.0202) + (A_663_ × 0.00802) × V/W, where V is the volume of DMSO and W corresponds to the fresh weight of the seedlings analyzed. Relative water content (RWC) was calculated as previously described (Smart and Bingham, 1974): RWC = (fresh weight – dry weight)/(turgid weight – dry weight). After floating harvested leaves on sterile distilled water for 6 h, their turgid weight was recorded. Leaves were weighed after drying in an oven at 85°C. All experiments were conducted at least twice, with three biological replicates for each treatment and five seedlings per replicate.

### Indirect measurement of fungal CO_2_ and its effect on plant growth

We used a tripartite Petri plate (Y-plate)-based assay (Kai and Piechulla, 2009) to determine whether CO_2_ produced by fungal culture contributed to plant growth. Cocultivation was performed as described above, except that only three *A. thaliana* seedlings per plate were grown due to space constraint. One compartment contained 8 mL 0.1 M Ba(OH)_2_, which reacts with CO_2_ to form BaCO_3_ precipitate. After 14 days of cocultivation, fresh shoot weight was measured, and the dry weight of BaCO_3_ was measured by filtering the solution through a filter paper followed by drying for 4 days at 50°C. Experiments were conducted twice, with three biological replicates.

### Gus staining

Histochemical staining of *in planta* GUS (β-glucuronidase) activity was performed as described in Jefferson et al. (1987). After cocultivation, *DR5::GUS* seedlings removed from MS were immersed in GUS staining solution, which consists of 50 mM sodium phosphate (pH 7.0), 10 mM EDTA (pH 8.0), 2 mM K_4_Fe(CN)_6_, 2 mM K_3_Fe(CN)_6_, 0.1% (v/v) Triton X-100, and 2 mM X-Gluc (5-bromo-4-chloro-3-indolyl-beta-D-glucuronic acid, cyclohexylammonium salt). After applying vacuum for 5 min, they were incubated overnight at 37°C in the dark with gentle agitation (75 rpm). These seedlings were washed sequentially with 70, 80, and 90% ethanol solutions until all chlorophylls were removed and were stored in 90% ethanol at 4°C until imaging. Staining patterns were observed using a stereomicroscope (Olympus SZ60, Center Valley, PA, USA) and imaged using the Olympus DP26 camera.

### NPA treatment

A stock solution of 1-naphthylthalamic acid (NPA; Sigma-Aldrich, St. Louis, MO, USA) in DMSO was used to produce MS containing 1, 5, and 10 μM NPA. Control plants were cultivated on MS containing DMSO only. A same volume of DMSO was used for all treatments. Experiments were conducted twice, with five seedlings per replicate.

### Analysis of *F. oxysporum* volatile compounds

Individual strains were cultured on half-strength PDA in 1 L glass flask for 7 days at room temperature. Charcoal purified air was introduced into these flasks and headspace volatiles were collected using SuperQ volatile collection trap (ARS Inc., Micanopy, FL, USA) at a rate of 0.5 L/min by applying vacuum for 24 h. Captured compounds were eluted using 120 μ L dichloromethane. As internal standards, 200 ng of octane and 200 ng of nonyl-acetate, dissolved in 5 μ L dichloromethane, were added to the elution. Eluted compounds were analyzed by gas chromatography-mass spectroscopy (GC-MS) in electron impact (EI) mode using a Hewlett-Packard 6890 GC equipped with an HP-5MS bonded phase capillary column (0.25 mm × 0.25 μm × 30 m; Agilent Technologies, Little Falls, DE, USA) interfaced to a HP 5973 MS (Hewlett-Packard, Palo Alto, CA, USA). The column temperature was programmed from an initial temperature of 40°C, with a 1 min hold time, 8°C min^−1^ to 240°C, and 40°C min^−1^ to 300°C with a 3-min hold at 300°C. Injections of 1 μ L were made with the inlet in splitless mode at 250°C with a split time of 0.75 min and helium carrier gas flow rate of 0.7 mLmin^−1^. EI analysis used the default settings (ion source: 230°C, quadrupole: 150°C, and with spectra generated at 70 eV), and identification was performed using retention indices, the NIST 08 and Adams 09 libraries, as well as authentic standards.

To determine the amount of ethylene produced by *F. oxysporum*, individual strains were cultured in 50 mL flask containing 25 mL half-strength Potato Dextrose Broth (PDB), on an orbital shaker (75 rpm) at room temperature for 3 days. Control was un-inoculated PDB. Prior to ethylene measurement, the flasks were opened inside a laminar hood for 5 min to release any previously produced ethylene, resealed with sterile rubber stopper and incubated for 24 h. A 1 mL of headspace air was collected from each flask using a sterile syringe, then injected into a GC with a flame ionization detector (HP 6890). Fungal biomass was measured after filtering the culture in each flask through 25 μ m filter paper (GE Healthcare Bio-Sciences, Pittsburgh, PA, USA) and dried at 65°C for 12 h. Ethylene production rate was calculated as ethylene produced (μ L per liter) per hour per g of fungal mycelia. The experiment was conducted twice, with three biological replicates for each strain.

### Statistical analysis

Collected data were analyzed by one-way ANOVA analysis using SAS 9.3 statistical analysis software (SAS Institute, Cary, NC). Means were compared using a Tukey's test and statistical significance was evaluated using the alpha value (α < 0.05).

## Results

### Volatile compounds produced by genetically and phenotypically diverse *F. oxysporum* strains enhanced the growth of *A. thaliana* and tobacco

We cocultivated 46 strains, representing 22 different *formae speciales* and phylogenetic lineages (O'Donnell et al., 2009), (Table 1) with *A. thaliana* ecotype Col-0 for 14 days to assess their ability to affect *A. thaliana* growth via volatile production (Figure 1). Compared with plants grown in the absence of fungal culture (control), 39 isolates (85%) significantly enhanced growth (Tukey's test with alpha value < 0.05). Two isolates, NRRL 32599 and FRC O-210, reduced shoot fresh weight by 50 and 25%, respectively, while five isolates caused no statistically significant effect. Two growth-enhancing isolates that exhibited the least variation, NRRL 26379 and NRRL 38335, and one neutral isolate, NRRL 38499, were chosen for further analyses.

**Figure 2:**
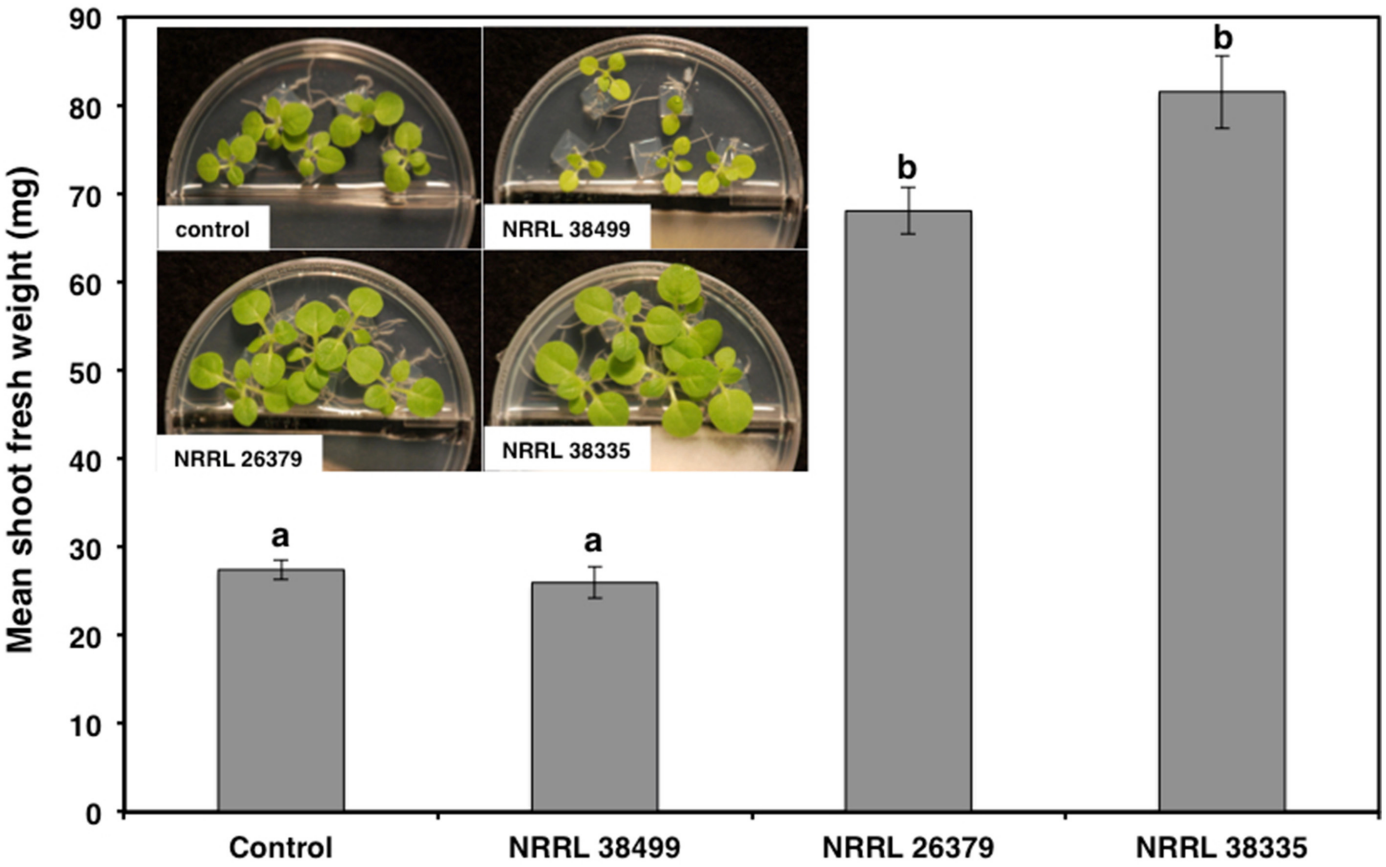
**Effect of *F. oxysporum* volatiles on *N. tabacum* growth**. Shoot fresh weight was measured after 2 weeks of cocultivation with no fungus (control), NRRL 38499, NRRL 26379, and NRRL 38335. Representative plates are shown in the insert. Means and standard errors for nine biological replicates per treatment, with five seedlings per replicate, are shown. Different letters on columns represent statistically significant differences by one-way analysis of variance.

**Figure 3:**
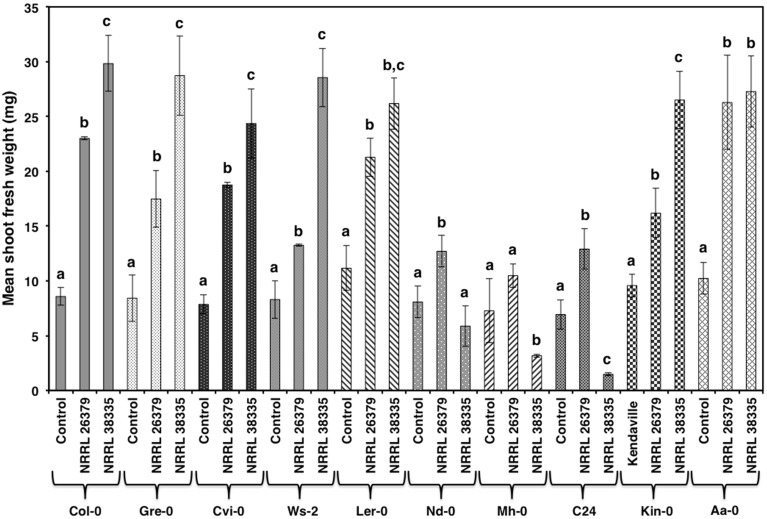
**Growth response of 10 *A. thaliana* ecotypes to *F. oxysporum* volatiles**. The mean shoot fresh weight of each ecotype was measured after 2 weeks of cocultivation with no fungus (control), NRRL 26379, and NRRL 38335. Means and standard errors for six biological replicates per treatment, with five seedlings per replicate, are shown. Different letters on columns represent statistically significant differences within each ecotype by one-way analysis of variance. Photos of ecotype C24 after this experiment are shown in Supplementary Figure 1.

As with Col-0, volatiles from NRRL 38499 did not enhance tobacco (*N. tabacum*) growth, but volatiles from NRRL 26379 and NRRL 38335 resulted in 2.5- and 3-fold shoot weight increases, respectively (Figure 2). Growth responses of nine additional *A. thaliana* ecotypes to volatiles from NRRL 26379 and NRRL 38335 were similar to those observed in Col-0 with a few exceptions (Figure 3). Ecotype C24 responded positively to NRRL 26379 but negatively to NRRL 38335 (Figure 3 and Supplementary Figure 1). Ecotype Mh-0 was neutral to NRRL 26379 volatiles, but volatiles from NRRL 38335 inhibited its growth, whereas Ecotype Nd-0 responded positively to NRRL 26379 but appeared neutral to NRRL 38335.

**Figure 4:**
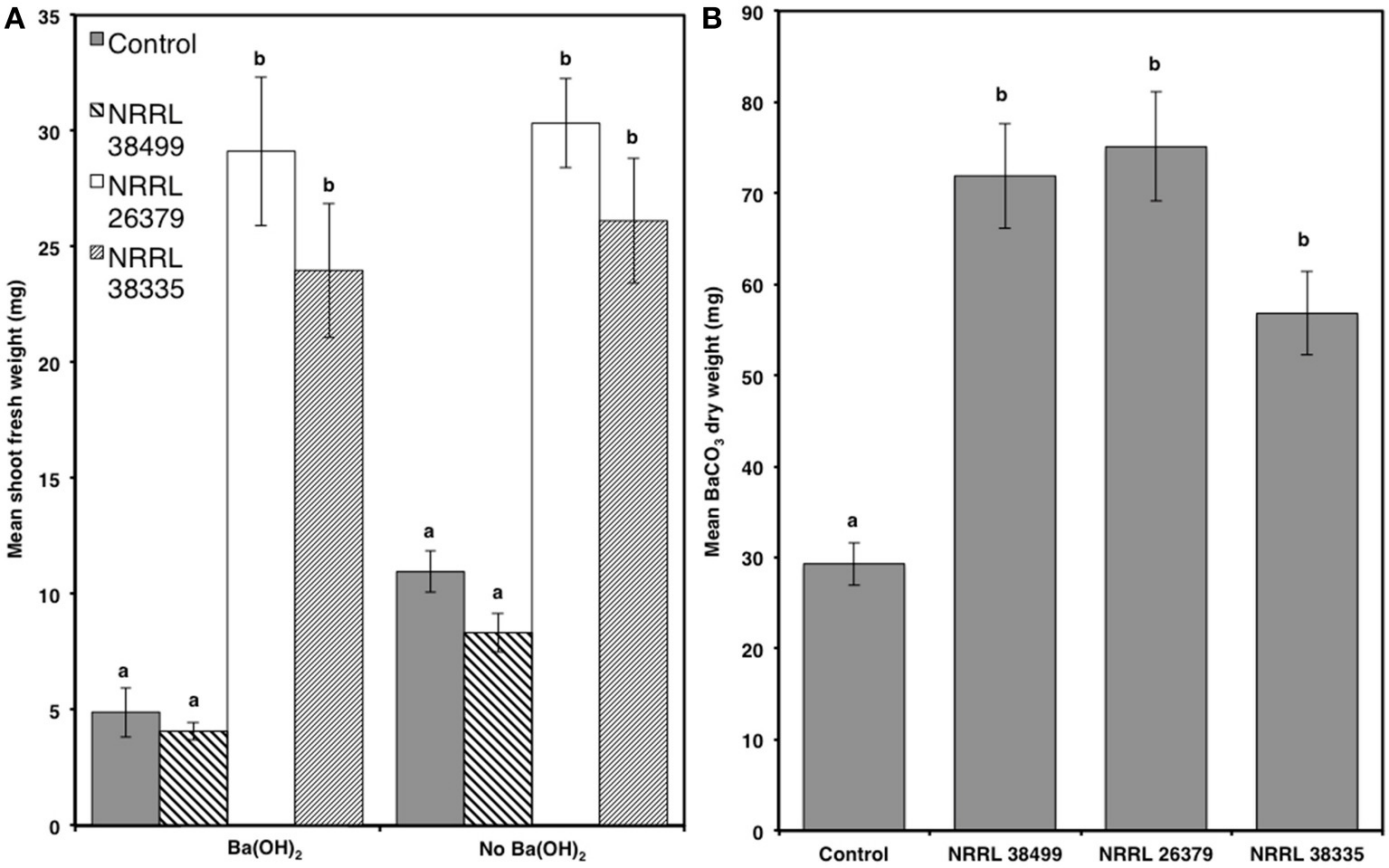
**Effect of CO_2_ produced by fungal culture on plant growth**. **(A)** Mean shoot fresh weights of Col-0 plants after 2 weeks of cocultivation with NRRL 38499, NRRL 26379, and NRRL 38335 in Y-plate in the presence and absence of Ba(OH)_2_ are shown. **(B)** Quantities of CO_2_ produced by these strains wereestimated by measuring the amount of dried BaCO_3_ precipitant formed during cocultivation. Means and standard errors for six biological replicates per treatment, with three seedlings per replicate, are shown. Different letters on columns represent statistically significant differences by one-way analysis of variance.

Since elevated atmospheric CO_2_ affects plant growth (Poorter and Navas, 2003; Baldwin, 2010), CO_2_ produced by microbial culture could also enhance plant growth (Kai and Piechulla, 2009). To determine whether CO_2_ produced by *F. oxysporum* culture contributed to plant growth, we compared Col-0 growth in the presence and absence of Ba(OH)_2_, which removes CO_2_ by forming BaCO_3_ precipitate (Kai and Piechulla, 2009). We also compared the amounts of CO_2_ produced by NRRL 38499, NRRL 26379, and NRRL 38335 by measuring the quantity of BaCO_3_ formed, and found no significant differences among them (Figure 4). Col-0 seedlings grown without fungal culture but in the presence of Ba(OH)_2_ displayed reduced shoot weight (by 55%) compared to those grown in the absence of Ba(OH)_2_. The magnitude of reduction was similar (51%) for those cocultivated with NRRL 38499, whereas only slight reduction was recorded in the seedlings cocultivated with NRRL 26379 and NRRL 38335 (Figure 4 and Supplementary Figure 2).

### *F. oxysporum* volatiles caused multiple changes in *A. thaliana*

To determine the time course of increase in shoot fresh weight, we harvested Col-0 seedlings after 5, 7, 10, and 14 days of cocultivation with NRRL 38499, NRRL 26379, and NRRL 38335 (Supplementary Figure 3). The growth response to NRRL 26379 and NRRL 38335 was not significantly different from that of the control and NRRL 38499 treatments up to day 10, but then growth of plants co-cultivated with NRRL 26379 and NRRL 38335 accelerated. To test if the degree of growth enhancement correlates with the duration of fungal volatile exposure, fungal culture was removed after cocultivation with these strains for 5, 7, and 10 days, but the seedlings were not harvested until day 14. For NRRL 38335, significant growth enhancement compared to control plants was noted at 7–10 days of cocultivation, while for NRRL 26379, it took 10–14 days of cocultivation (Supplementary Figure 4).

**Figure 5:**
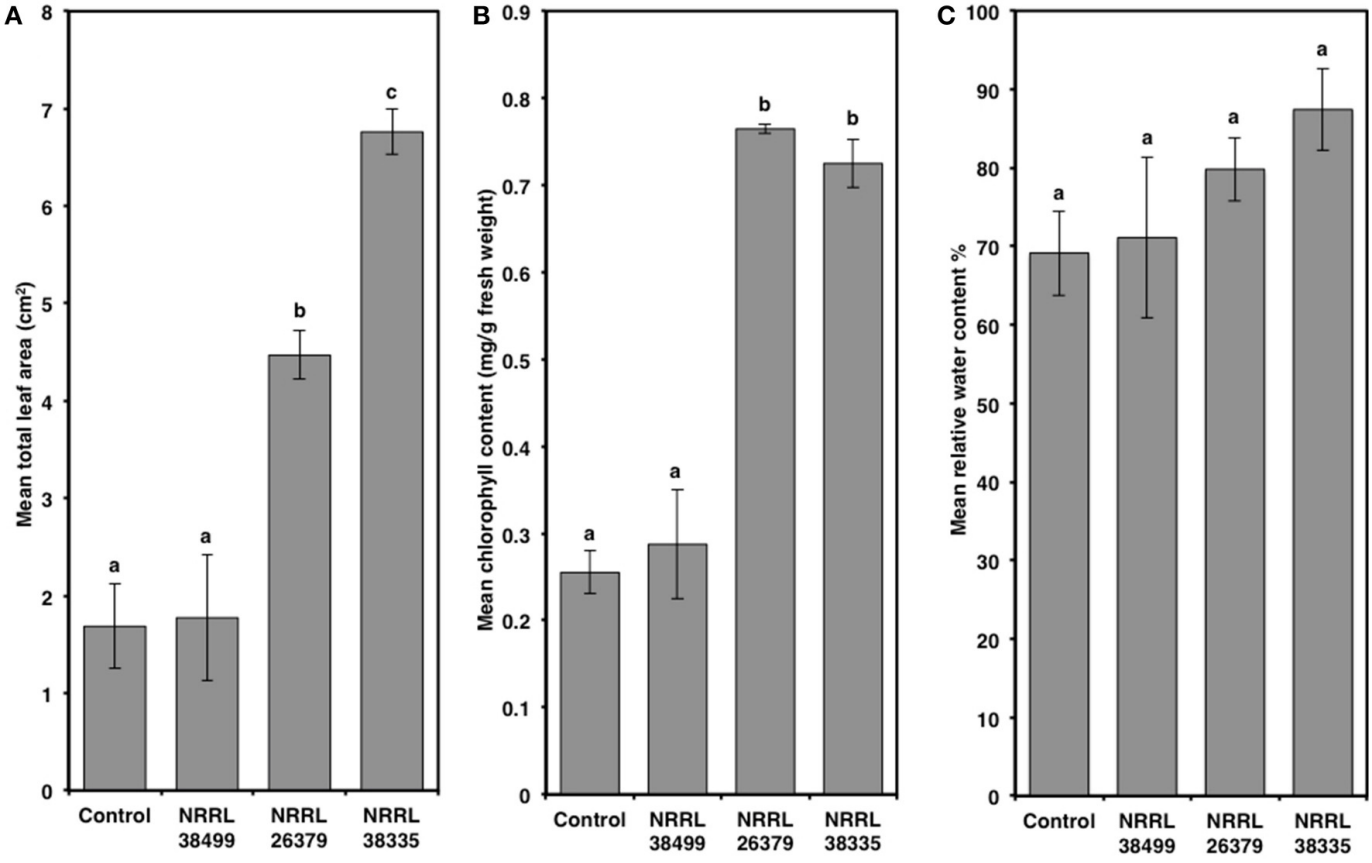
**Alterations of leaves caused by *F. oxysporum* volatiles**. **(A)** total leaf area, **(B)** chlorophyll content, and **(C)** relative water content in Col-0 leaves after 2 weeks of cocultivation with no fungus (control), NRRL 38499, NRRL 26379, and NRRL 38335 are shown. Means and standard errors for four biological replicates per treatment, with five seedlings per replicate, are shown. Different letters on columns represent statistically significant differences by one-way analysis of variance. Scanned images of representative leaves used for **(A)** are shown in Supplementary Figure 5.

**Figure 6:**
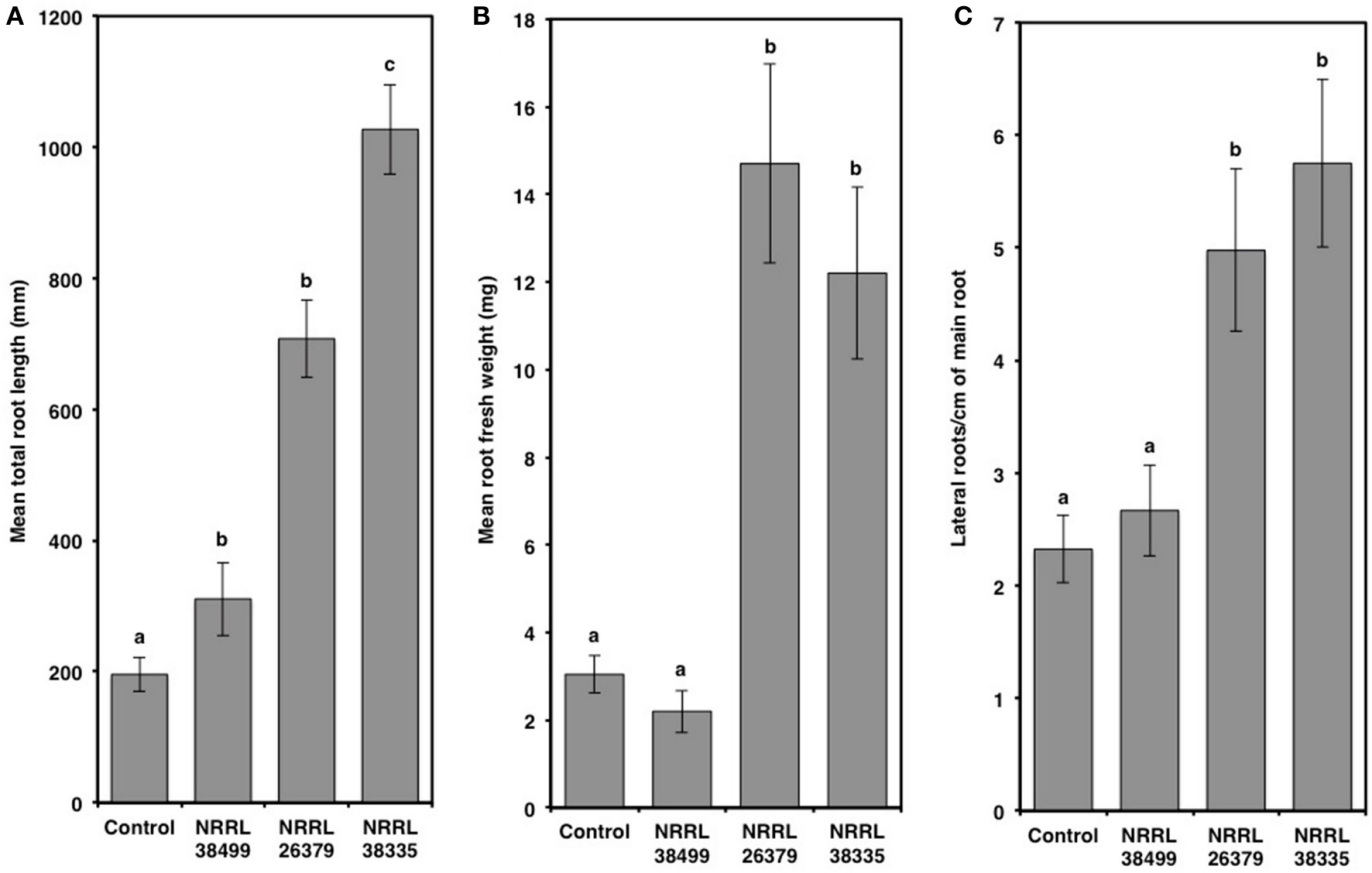
**Root alterations caused by *F. oxysporum* volatiles**. **(A)** total root length, **(B)** root fresh weight, and **(C)** lateral root density of Col-0 were quantified after 14 days of cocultivation with no fungus (control), NRRL 38499, NRRL 26379, and NRRL 38335. Means and standard errors for three biological replicates per treatment, with five seedlings per replicate, are shown. Different letters on columns represent statistically significant differences by one-way analysis of variance. Supplementary Figure 6 shows representative roots collected after this experiment.

Compared to control plants, cocultivation with NRRL 26379 and NRRL 38335 increased the total leaf area by 2.7- and 4-fold, respectively, and the chlorophyll content by 3-fold (Figure 5 and Supplementary Figure 5). However, RWC did not significantly differ amongst the treatments (Tukey's test with alpha value < 0.05). Cocultivation with NRRL 26379 and NRRL 38335 also increased the mass (4.8- and 4-fold, respectively) and total length (3.6- and 5.2-fold, respectively) of roots (Figure 6 and Supplementary Figure 6). The lateral root density was more than doubled (Figure 6). The root to shoot ratio in plants cocultivated with NRRL 26379 and NRRL 38335 nearly doubled compared to control plants.

### Growth responses of *A. thaliana* hormone signaling mutants suggest the involvement of auxin signaling in mediating growth enhancement by *F. oxysporum* volatiles

**Figure 7:**
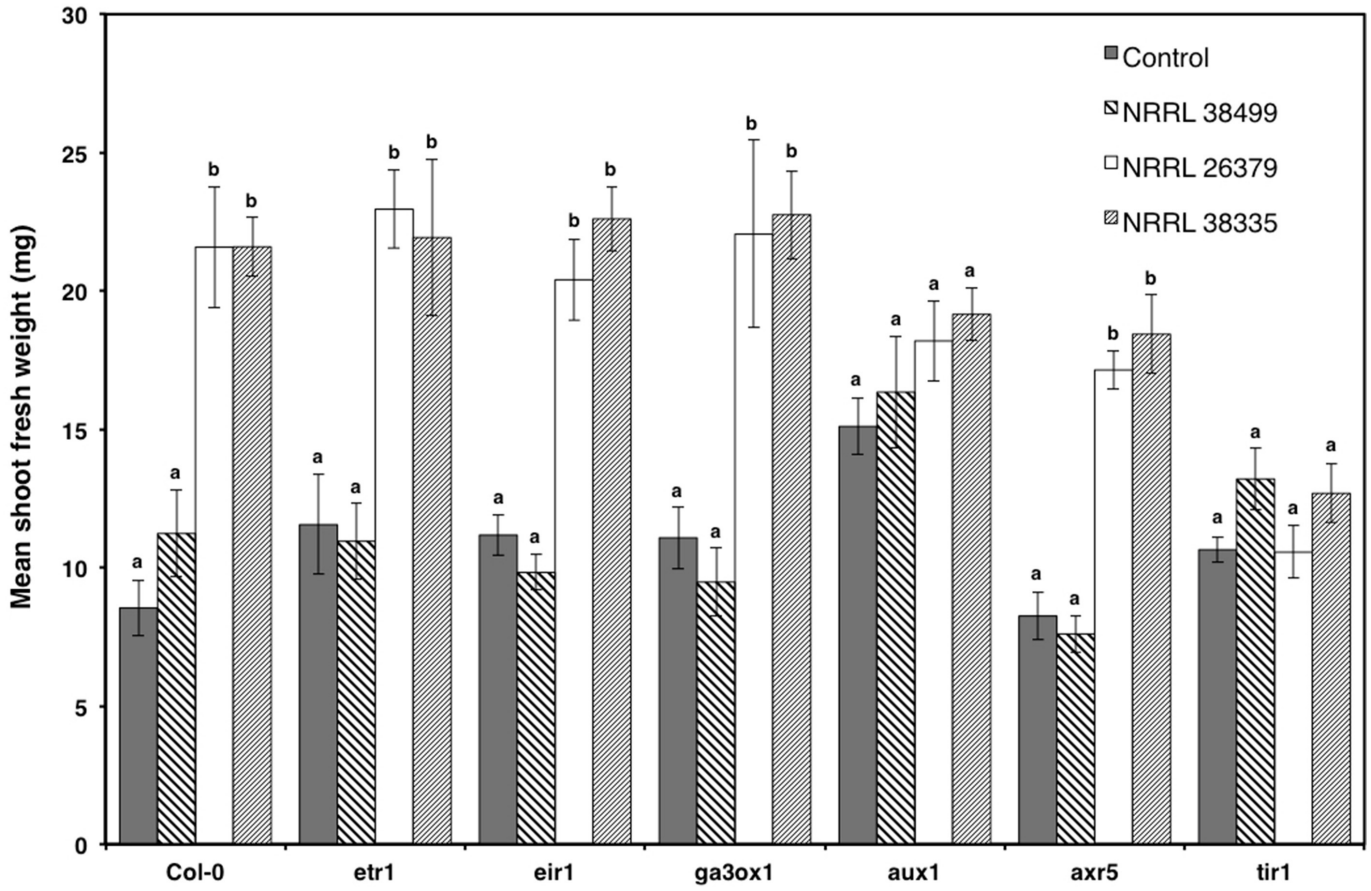
**Growth response of *A. thaliana* hormone signaling mutants to *F. oxysporum* volatiles**. Growth response of *six* mutants to volatiles produced by NRRL 38499, NRRL 26379, and NRRL 38335 are shown. Means and standard errors for two biological replicates per treatment, with five seedlings per replicate, are shown. Different letters on columns represent statistically significant differences within each mutant line by one-way analysis of variance.

To elucidate the mechanism underlying *F. oxysporum* volatile-mediated growth enhancement, selected *A. thaliana* mutants that are defective in hormone signaling were exposed to *F. oxysporum* volatiles (Figure 7). Based on a previous study showing the involvement of auxin in bacterial volatile-mediated plant growth enhancement (Zhang et al., 2007), most of the chosen mutants are defective in auxin signaling. The *ETR1* gene encodes an ethylene receptor (Schaller et al., 1995; Chen et al., 2002). The *EIR1* gene, encoding a root specific auxin efflux carrier, is involved in gravitropism (Luschnig et al., 1998). The *AXR5* and *AUX1* genes encode a repressor of auxin response (Yang et al., 2004) and an auxin influx transporter (Yang et al., 2006), respectively. The *TIR1* gene encodes an F-box protein, a component of the ubiquitin ligase complex that degrades the AUX/IAA transcriptional repressor proteins, and is thus required for activating auxin-responsive genes (Dharmasiri et al., 2005; Kepinski and Leyser, 2005). The *GA3OX1* gene product catalyzes the first step in gibberellic acid biosynthesis (Mitchum et al., 2006). The mutants *etr1, eir1, ga3ox1*, and *axr5* looked comparable to Col-0, upon cocultivation with NRRL 26379 and NRRL 38335 (Figure 7). However, *aux1* and *tir1* did not exhibit significant growth enhancement (Figure 7).

**Figure 8:**
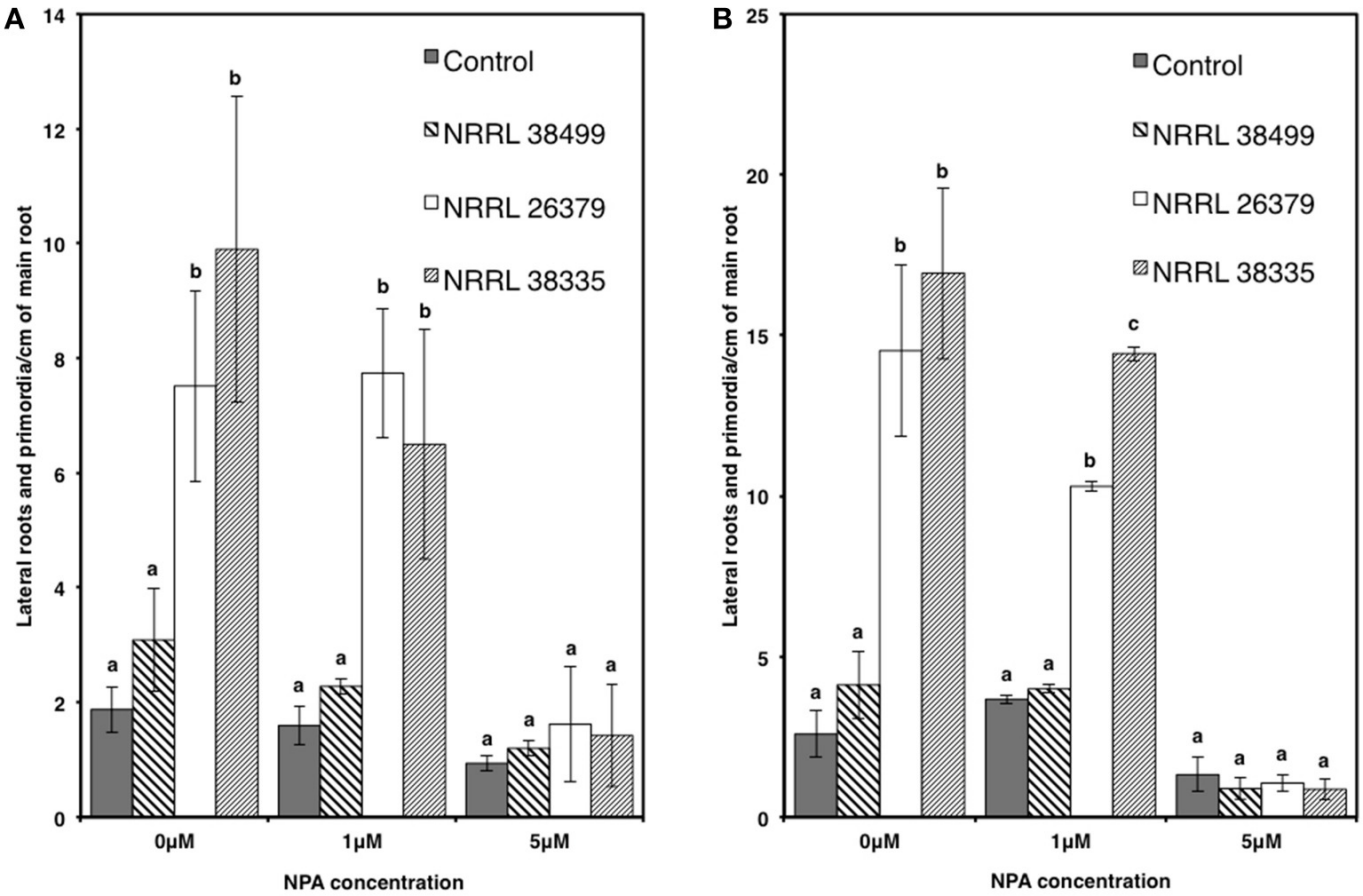
**Effect of *F. oxysporum* volatiles on *A. thaliana* lateral root development in the presence of NPA**. Lateral root density (both lateral roots and lateral root primordia) of Col-0 containing *DR5::GUS* in the presence of 0, 1, and 5 μ M NPA was determined after cocultivation with no fungus (control), NRRL 38499, NRRL 26379, and NRRL 38335 for 7 **(A)** and 14 **(B)** days. Means and standard errors for two biological replicates per treatment, with five seedlings per replicate, are shown. Different letters on columns denote statistically significant differences within each treatment by one-way analysis of variance.

**Figure 9:**
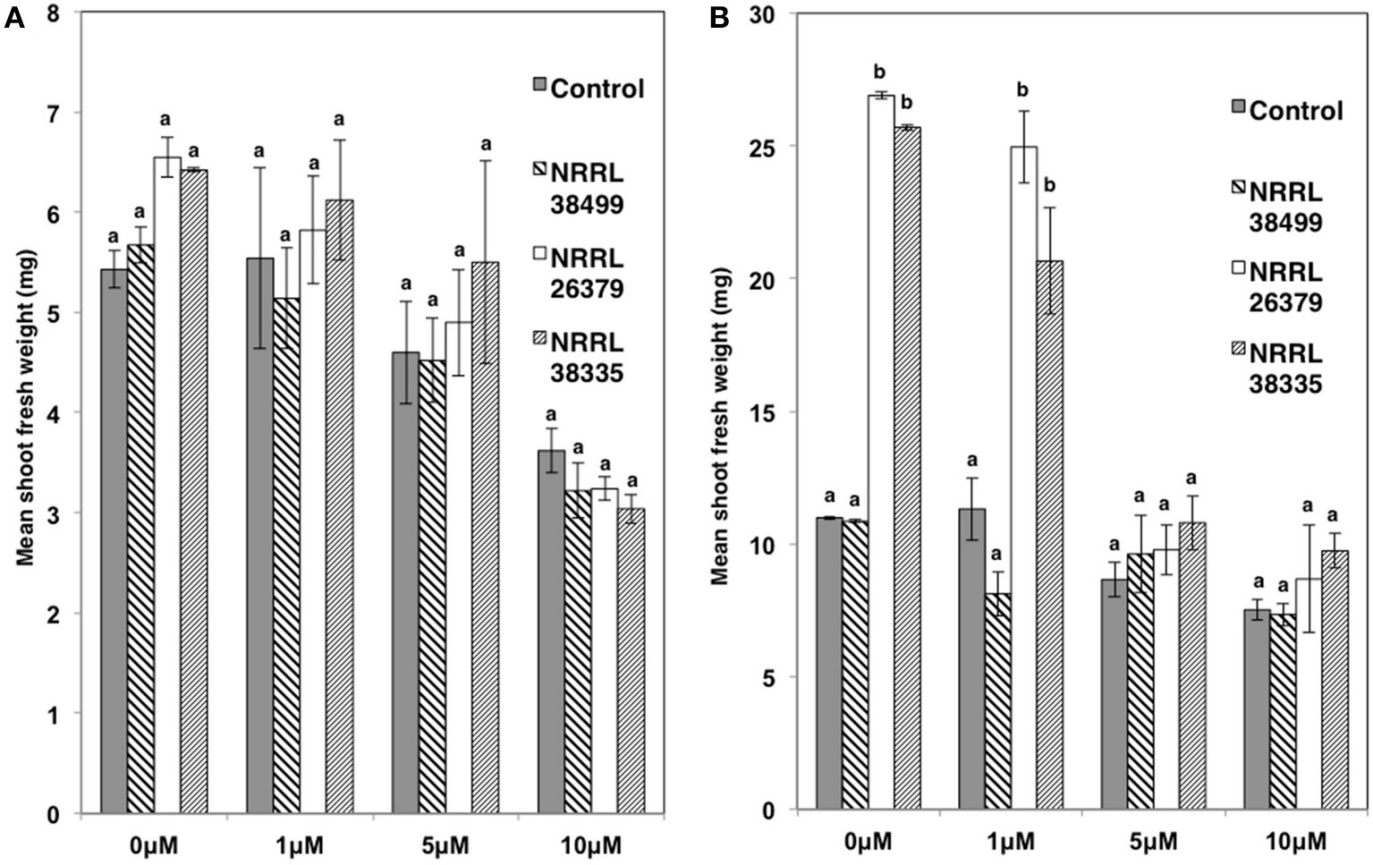
**Effect of NPA on volatile-mediated growth enhancement**. The shoot fresh weight of Col-0 containing *DR5::GUS* was measured after cocultivation with no fungus (control), NRRL 38499, NRRL 26379, and NRRL 38335 for 7 **(A)** and 14 **(B)** days in the presence of NPA. Means and standard errors for three biological replicates per treatment, with five seedlings per replicate, are shown. Different letters on columns denote statistically significant differences within each NPA treatment by one-way analysis of variance.

We also used transgenic Col-0 carrying the *DR5::GUS* reporter, which has been widely used for monitoring the spatial and temporal patterns of auxin response (Dubrovsky et al., 2008; Lucas et al., 2008; Péret et al., 2009). The stain permitted easy counting of lateral root tips and primordia. Consistent with the data shown in Figure 6, the density of lateral roots in *DR5::GUS* plants became significantly higher when cocultivated with NRRL 26379 and NRRL 38335 for 7 and 14 days (Figure 8 and Supplementary Figure 7). GUS activity was primarily localized at root tips and lateral root primordia in all treatments, but the magnitude of resulting GUS staining varied among strains (Supplementary Figures 7, 8). At 7 days after cocultivation with NRRL 26379 and NRRL 38335, straining was stronger and more extensive than that observed in plants cocultivated with no fungus and NRRL 38499 (Supplementary Figure 8). However, at 14 days after cocultivation, no obvious difference was observed between these groups. Shoot growth, root development and GUS activity were also analyzed in the presence of different concentrations of 1-naphthylphthalamic acid (NPA), an auxin efflux inhibitor (Keller et al., 2004). In the presence of 1 μ M NPA, the lateral root density of plants cocultivated with NRRL 26379 and NRRL 38335 still remained significantly higher than that observed in the other two treatments at both days 7 and 14 (Figure 8 and Supplementary Figure 9). However, in the presence of 5 μM NPA, lateral root formation was drastically reduced in all treatments, and GUS staining was reduced compared to that observed with no or 1 μ M NPA (Figure 8 and Supplementary Figure 9). Growth enhancement by NRRL 26379 and NRRL 38335 volatiles was not affected by 1 μ M NPA, but at higher concentrations (5 and 10 μ M), the growth enhancement effect was abolished (Figure 9). We also determined whether growth enhancement of tobacco plants by *F. oxysporum* volatiles was affected by NPA (Supplementary Figure 10). Although tobacco plants were less sensitive to NPA than *A. thaliana*, in the presence of 5 and 10 μ M NPA, growth enhancement by NRRL 26379 and NRRL 38335 volatiles was significantly reduced.

### Volatile compounds produced by *F. oxysporum*

**Figure 10:**
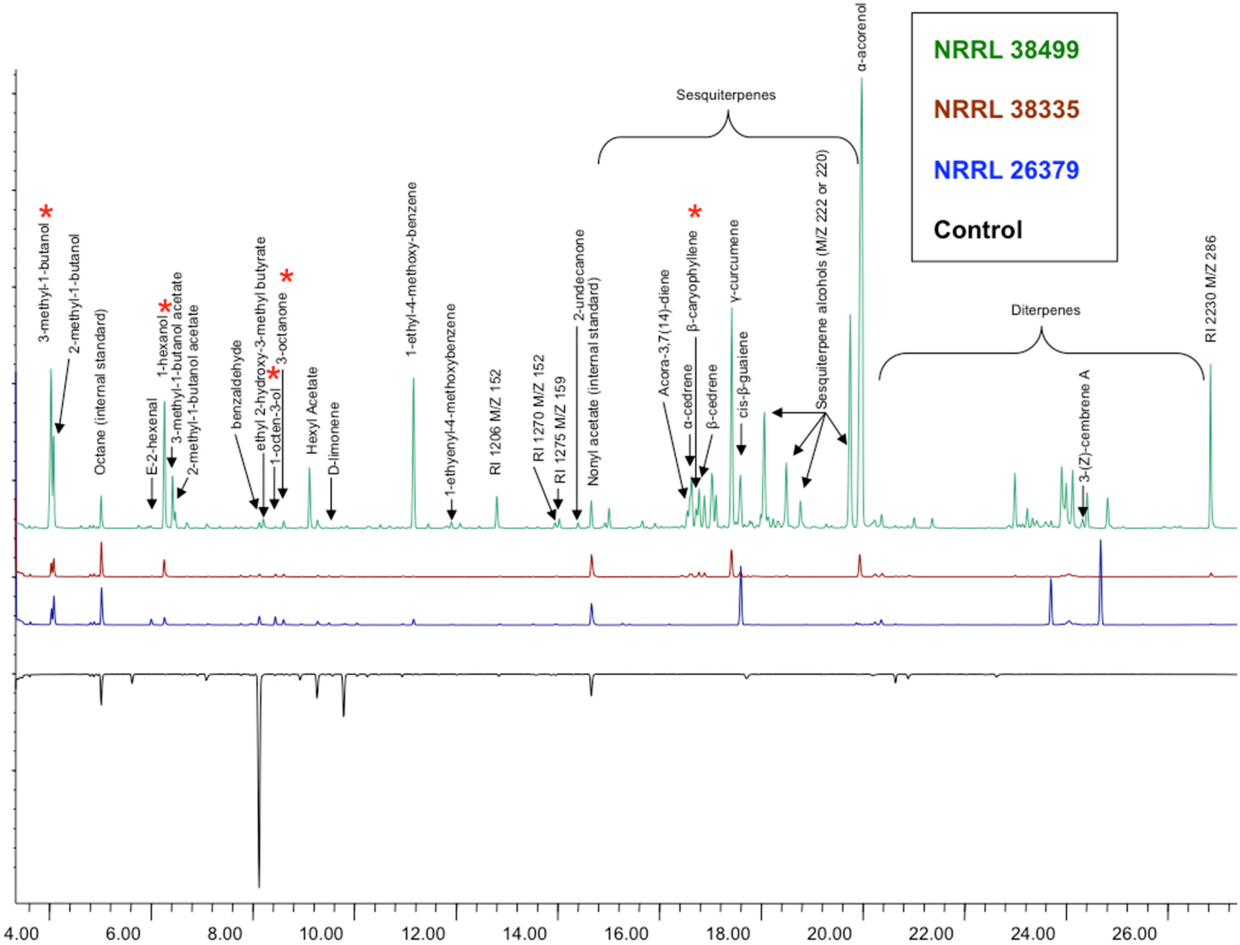
**Volatile compounds produced by *F. oxysporum***. Headspace volatiles from no fungus (bottom, control), NRRL 38499, NRRL 26379, and NRRL 38335 cultured on PDA were analyzed using GC-MS. Asterisks denote compounds that have been shown to affect plants in previously published studies.

Analysis of volatile compounds produced by NRRL 38499, NRRL 26379 and NRRL 38335, using GC-MS, led to the tentative identification of several compounds (Table 2 and Figure 10), but many others remain to be identified. Interestingly, NRRL 38499, which did not enhance plant growth, produced more diverse compounds and higher quantities of many compounds than NRRL 26379 and NRRL 38335. The most abundant classes of volatile compounds were sesquiterpenes and diterpenes, most of which remain unidentified. NRRL 38499 produced many more sesquiterpenes and diterpenes than NRRL 26379 or NRRL 38335. All three isolates produced compounds that have been shown to inhibit plant growth, such as 3-methyl-1-butanol, 1-hexanol, 1-octen-3-ol and 3-octanone. Even though no compounds uniquely produced by both NRRL 26379 and NRRL 38335 were detected, NRRL 26379 produced 1-2 diterpenes that appeared absent in NRRL 38335. These patterns suggested the possibility that all three strains produced plant growth enhancing volatile(s), but NRRL 38499 might also produce compounds that inhibit plant growth, thus negating growth enhancement effect or even stunting plant growth. Comparison of ethylene production among the three strains did not reveal significant differences (Supplementary Figure 11), suggesting that ethylene is not one such compound.

**Table 2:** **Identified *F. oxysporum* volatile compounds**.

**Volatile compounds**		**Strains**	
	**NRRL 38499**	**NRRL 38335**	**NRRL 26379**
^*^3-methyl-1-butanol	✓	✓	✓
2-methyl-1-butanol	✓	✓	✓
E-2-hexanal	✓	✓	✓
^*^1-hexanol	✓	✓	✓
3-methyl-1-butanol acetate	✓	✕	✕
2-methyl-1-butanol acetate	✓	✕	✕
Benzaldehyde	✓	✓	✓
ethyl 2-hydroxy-3-methyl butyrate	✓	✕	✕
^*^1-octen-3-ol	✓	✓	✓
^*^3-octanone	✓	✓	✓
Hexyl Acetate	✓	✕	✕
D-limonene	✕	✓	✓
1-ethyl-4-methoxy-benzene	✓	✓	✓
1-ethyenyl-4-methoxybenzene	✓	✕	✕
2-undecanone	✓	✕	✕
Acora-3,7 (14)-diene	✓	✕	✕
α-cedrene	✓	✓	✕
^*^β-caryophylene	✓	✓	✕
β-cedrene	✓	✓	✕
γ-curcumene	✓	✓	✕
cis-β-guaiene	✓	✓	✓
3-(Z)-cembrene A	✓	✕	✕

*✓ and ✕ denote the presence and absence, respectively, of each compound*.

* *Compounds with known/suspected effect on plant growth*.

## Discussion

Both plants and microbes employ multiple strategies to coordinate or take control of their interactions, resulting in diverse forms of association ranging from mutualistic to pathogenic. One strategy that has evolved convergently in multiple microbial kingdoms is secreting or injecting a variety of effectors that manipulate plant defense machineries. Secretion of effectors is not limited to pathogens, e.g., symbiotic associations also require suppression of host defense by microbial partners (Evangelisti et al., 2014). Effectors include proteins (Deslandes and Rivas, 2012; Okmen and Doehlemann, 2014; Rovenich et al., 2014), metabolites (Collemare and Lebrun, 2011), and nucleic acids (Weiberg et al., 2014), but compared to research on protein effectors, work on the other types of effectors lags considerably behind. In addition to effectors, microbes also secrete various molecules that affect plant health both directly and indirectly (Bonfante and Anca, 2009; Bednarek et al., 2010), such as siderophores for iron acquisition, enzymes for nutrient mobilization and antibiotics. Because volatile metabolites can travel through air and porous soils, they can potentially function as signals for both short- and long-range organismal interactions even in the absence of water as medium. We tested the hypothesis that certain volatiles produced by plant-associated fungi affect their interaction with plants.

### Multifaceted roles of *F. oxysporum* volatiles?

Potential benefits of producing volatiles that enhance plant growth are likely to be found in *F. oxysporum*'s main ecological niches, the rhizosphere and within plants. The rhizosphere offers rich nutrient sources for microflora (e.g., root exudates, mucilage, dead root cells). Accordingly, the rhizosphere harbors diverse groups of microbes and serves as their “playground and battlefield” (de Weert and Bloemberg, 2006; Raaijmakers et al., 2009). Due to competition among rhizosphere dwellers and antimicrobials released by plants, the membership to this community (i.e., rhizosphere competency) requires a few prerequisites (de Weert and Bloemberg, 2006). The frequent presence of *F. oxysporum* in the rhizosphere of diverse plants, including those that are not considered as their hosts (Malcolm et al., 2013), indicates its broad rhizosphere competency. In this study we showed that volatiles from genetically and ecologically diverse strains (Table 1) significantly enhanced plant growth (Figures 1–3). This ability likely contributes to its rhizosphere competency by increasing nutrient availability, since enhanced root growth (e.g., Figure 6) would be expected to increase plant-derived exudates as well as root tissue for potential colonization.

Another potential benefit comes from the root developmental change caused by its volatiles. Volatiles from NRRL 26379 and NRRL 38335 not only increased the root biomass but also doubled the density of lateral roots (Figures 6, 8 and Supplementary Figure 7). Root penetration by *F. oxysporum* occurs primarily through the meristematic region of primary and lateral roots (Bishop and Cooper, 1983; Turlier et al., 1994; Czymmek et al., 2007). For pathogenic strains, increased formation of lateral roots will likely facilitate infection by presenting more potential entry points to the vascular system. NRRL 38272, a previously characterized pathogenic strain of *A. thaliana* (Ospina-Giraldo et al., 2003; Czymmek et al., 2007), as well as most other strains that are likely to be pathogenic to crucifer plants (formae speciales *conglutinans, mathioli*, and *raphani*), enhanced *A. thaliana* growth (Figure 1). Since the integrity of adjacent cortical cells is disrupted during hyphal growth of *F. oxysporum* through the vascular system (Czymmek et al., 2007), another possible role of volatile compounds is to act as phytotoxic virulence factors.

### Which compounds affect plant growth?

Volatile profiles (Figure 10 and Table 2) present an enigma, since both growth-promoting and growth-inhibiting compounds were detected, along with compounds with unknown effects. NRRL 38499, but not growth-enhancing strains NRRL 26379 and NRRL 38335, seems to produce β-caryophyllene, a sesquiterpene that was previously reported to enhance lettuce growth (Minerdi et al., 2011). In addition, NRRL 38499 produced more compounds in both the number and quantity than NRRL 26379 and NRRL 38335, and no compounds appear unique to the latter two. These patterns raised the possibility that NRRL 38499 might produce both growth-inhibitory and -stimulatory compounds, thus making their net effect on plants neutral. Although specific compound(s) that are inhibitory remain to be confirmed, previous studies suggest some candidates, such as 3-methyl-1-butanol, 1-hexanol, 1-octen-3-ol, and 3-octanone (Figure 10). Volatiles produced by truffles, which inhibited *A. thaliana* growth, included these compounds, and their synthetic versions also inhibited *A. thaliana* growth (Splivallo et al., 2007). Additional candidates include volatile terpenes. Some terpenes have been shown to inhibit the growth of other organisms, including plants (Collado et al., 2007; Kramer and Abraham, 2012). More abundant production of volatile sesquiterpenes and diterpenes by NRRL 38499 than NRRL 26379 and NRRL 38335 (Figure 10) suggests that some of these terpenes may inhibit plant growth. An attempt to test this supposition by disrupting the production of these groups of terpenes in NRRL 38499 via targeted mutagenesis of their biosynthetic genes is in progress.

It was suggested that promotion of plant growth by microbial volatiles could simply be a result of CO_2_ enrichment during cocultivation (Kai and Piechulla, 2009). We found no significant difference in CO_2_ production among growth-promoting and neutral strains of *F. oxysporum*. Removal of CO_2_ using Ba(OH)_2_ reduced the growth of *A. thaliana* without fungus and cocultivated with NRRL 38499 (Figure 4), indicating that carbon fixation via photosynthesis still limits plant growth even when plants are cultured on sucrose-containing medium. However, no significant reduction in growth was observed for plants cocultivated with NRRL 26379 and NRRL 38335 (Figure 4), arguing against the proposition that increased CO_2_ production solely drove plant growth enhancement. Higher chlorophyll in the plants exposed to volatiles from NRRL 26379 and NRRL 38335, but not by volatiles from NRRL 38499 suggests more active photosynthesis in plants cocultivated with the growth-promoting strains.

### Mechanism underpinning growth enhancement by *F. oxysporum* volatiles

Manipulation of auxin transport and signaling by *F. oxysporum* volatiles seems to underpin growth enhancement (Figures 7–9 and Supplementary Figures 7–9). Mutation of either *AUX1*, encoding an auxin influx transporter (Yang et al., 2006) or *TIR1*, encoding an auxin receptor that regulates the expression of auxin-responsive genes (Dharmasiri et al., 2005; Kepinski and Leyser, 2005), negated growth promotion by *F. oxysporum* volatiles (Figure 7). In addition, chemical inhibition of auxin efflux blocked volatile-mediated growth promotion in both *A. thaliana* and tobacco (Figures 8, 9). Temporal and spatial patterns of GUS activity in *DR5::GUS* plants indicated earlier (before shoot biomass enhancement became visible) and stronger activation of lateral root development via auxin signaling (Supplementary Figures 7–9). Volatiles from other microbes that enhanced plant growth similarly affected root growth and development (Zhang et al., 2007; Gutierrez-Luna et al., 2010; Hung et al., 2013). *Bacillus subtilus* volatile-mediated growth enhancement of *A. thaliana* was also blocked by NPA (Zhang et al., 2007), suggesting that manipulation of auxin transport and signaling using volatiles is a mechanism employed by diverse microbes. As discussed above, this manipulation likely contributes to the fitness of plant-associated microbes.

Kidd et al. (2011) showed that several genes involved in auxin signaling were differentially regulated in response to *F. oxysporum* infection. In addition, mutants in some of these genes, as well as several other mutants defective in auxin transport or signaling, were more resistant to *F. oxysporum*. Inhibition of auxin efflux by NPA and 2,3,5-trioidobenzoic acid also enhanced resistance. Collectively, these results suggest that *F. oxysporum* exploits auxin transport and signaling to facilitate infection in *A. thaliana*. Clearly, *F. oxysporum* volatiles are candidate signals for this manipulation.

## Future directions

Exploration of plant-microbe communication through volatile compounds will likely reveal novel mechanisms underpinning plant-microbe interactions and offer potential applications (Morath et al., 2012; Bitas et al., 2013). Given the vast diversity of fungi associated with plants and their seemingly critical, yet mostly underexplored, roles in plant health, concerted efforts are needed to understand if and how volatiles produced by such fungi affect plant growth and how such molecules are perceived and processed by plants. Another key question is whether volatiles from *F. oxysporum* and other fungi also affect other traits. Several studies showed that volatiles produced by several bacterial species enhanced both plant growth and stress resistance (Bitas et al., 2013). Since increased plant growth supports microbial growth in the rhizosphere, enhanced resistance to biotic and abiotic stress also provides improves potential habitat for the fungus under unfavorable environmental conditions. In addition, as discussed above, manipulation of plant growth and development via volatile production may also facilitate infection. Identification of specific volatile compound(s) that affect plant growth and their biosynthetic pathways in *F. oxysporum* is a key step to address these questions. Exposure of plants to synthetic versions of candidate compounds has been employed to identify bacterial and fungal volatiles compounds that enhance plant growth (e.g., Ryu et al., 2003; Splivallo et al., 2007). Although this approach is not sufficient for proving their involvement in affecting plant growth in the field, it helps identify candidate compounds for further research. Knowledge of the identity and biosynthetic pathways for fungal volatile semio-chemicals, in combination with targeted genetic manipulation, will help confirm their involvement in plant growth via disruption of their biosynthesis. Ryu et al. (2003) used this approach to show the involvement of two compounds, acetoin and 2,3-butanediol, in causing growth enhancement by *Bacillus* spp.

### Conflict of interest statement

The authors declare that the research was conducted in the absence of any commercial or financial relationships that could be construed as a potential conflict of interest.

## References

[B1] Baldwin I. T. Kessler A. Halitschke R. (2002). Volatile signaling in plant-plant-herbivore interactions: what is real? Curr. Opin. Plant Biol. 5, 351–354. 10.1016/S1369-5266(02)00263-7 12179970

[B2] Baldwin I. T. (2010). Plant volatiles. Curr. Biol. 20, 392–397. 10.1016/j.cub.2010.02.052 20462477

[B3] Bednarek P. Kwon C. Schulze-Lefert P. (2010). Not a peripheral issue: secretion in plant-microbe interactions. Curr. Opin. Plant Biol. 13, 378–387. 10.1016/j.pbi.2010.05.002 20558098

[B4] Bennett J. W. Hung R. Lee S. Padhi S. (2012). Fungal and bacterial volatile organic compounds: an overview and their role as ecological signaling agents, in The Mycota IX Fungal Interactions, ed Hock B. (Berlin-Heidelberg; Springer), 229–250.

[B5] Bishop C. D. Cooper R. M. (1983). An ultrastructural study of vascular colonization in 3 vascular wilt diseases. 1. Colonization of susciptible cultivars. Physiol. Plant Pathol. 23, 323–343. 10.1016/0048-4059(83)90018-8

[B6] Bitas V. Kim H. S. Bennett J. W. Kang S. (2013). Sniffing on microbes: diverse roles of microbial volatile organic compounds in plant health. Mol. Plant Microbe Interact. 26(8):835–843. 10.1094/MPMI-10-12-0249-CR 23581824

[B7] Bleecker A. B. Estelle M. A. Somerville C. Kende H. (1988). Insensitivity to ethylene confered by a dominant mutation in *Arabidopsis thaliana*. Science 241, 1086–1089. 10.1126/science.241.4869.1086 17747490

[B8] Bonfante P. Anca I. A. (2009). Plants, mycorrhizal fungi, and bacteria: a network of interactions. Annu. Rev. Microbiol. 63, 363–383. 10.1146/annurev.micro.091208.073504 19514845

[B9] Chang C. Kwok S. F. Bleecker A. B. Meyerowitz E. M. (1993). *Arabidopsis* ethylene-response gene ETR1 similarity of product to 2-component regulators. Science 262, 539–544. 10.1126/science.8211181 8211181

[B10] Chen Y. F. Randlett M. D. Findell J. L. Schaller G. E. (2002). Localization of the ethylene receptor ETR1 to the endoplasmic reticulum of Arabidopsis. J. Biol. Chem. 277, 19861–19866. 10.1074/jbc.M201286200 11916973

[B11] Collado I. G. Sánchez A. J. M. Hanson J. R. (2007). Fungal terpene metabolites: biosynthetic relationships and the control of the phytopathogenic fungus *Botrytis cinerea*. Nat. Prod. Rep. 24, 674–686. 10.1039/b603085h 17653354

[B12] Collemare J. Lebrun M.-H. (2011). Fungal secondary metabolites: Ancient toxins and novel effectors in plant–microbe interactions, in Effectors in Plant–Microbe Interactions, eds Martin F. Kamoun S. (Oxford, UK:Wiley & Sons), 377–400.

[B13] Czymmek K. J. Fogg M. Powell D. H. Sweigard J. Park S.-Y. Kang S. (2007). *In vivo* time-lapse documentation using confocal and multi-photon microscopy reveals the mechanisms of invasion into the *Arabidopsis* root vascular system by *Fusarium oxysporum*. Fungal Genet. Biol. 44, 1011–1023. 10.1016/j.fgb.2007.01.012 17379550

[B14] de Weert S. Bloemberg G. V. (2006). Rhizosphere competence and the role of root colonization in biocontrol, in Plant-Associated Bacteria, ed Gnanamanickam S. S. (Berlin, Heidelberg; Springer), 317–333.

[B15] Deslandes L. Rivas S. (2012). Catch me if you can: bacterial effectors and plant targets. Trends Plant Sci. 17, 644–655. 10.1016/j.tplants.2012.06.011 22796464

[B16] Dharmasiri N. Dharmasiri S. Estelle M. (2005). The F-box protein TIR1 is an auxin receptor. Nature 435, 441–445. 10.1038/nature03543 15917797

[B17] Dubrovsky J. G. Sauer M. Napsucialy-Mendivil S. Ivanchenko M. G. Friml J. Shishkova S.. (2008). Auxin acts as a local morphogenetic trigger to specify lateral root founder cells. Proc. Natl. Acad. Sci. U.S.A. 105, 8790–8794. 10.1073/pnas.0712307105 18559858 PMC2438385

[B18] Evangelisti E. Rey T. Schornack S. (2014). Cross-interference of plant development and plant-microbe interactions. Curr. Opin. Plant Biol. 20, 118–126. 10.1016/j.pbi.2014.05.014 24922556

[B19] Gutierrez-Luna F. M. Lopez-Bucio J. Altamirano-Hernandez J. Valencia-Cantero E. de la Cruz H. R. Macias-Rodriguez L. (2010). Plant growth-promoting rhizobacteria modulate root system architecture in *Arabidopsis thaliana* through volatile organic compound emission. Symbiosis 51, 75–83. 10.1007/s13199-010-0066-2

[B20] Han S. H. Lee S. J. Moon J. H. Park K. H. Yang K. Y. Cho B. H.. (2006). GacS-dependent production of 2R, 3R-butanediol by *Pseudomonas chlororaphis* O6 is a major determinant for eliciting systemic resistance against *Erwinia carotovora* but not against *Pseudomonas syringae* pv. tabaci in tobacco. Mol. Plant Microbe Interact. 19, 924–930. 10.1094/MPMI-19-0924 16903358

[B21] Herrmann A. (2010). The Chemistry and Biology of Volatiles. Chichester, UK:Wiley & Sons.

[B22] Hiscox J. D. Israelstam G. F. (1979). A method for the extraction of chlorophyll from leaf tissue without maceration. Can. J. Bot. 57, 1332–1334. 10.1139/b79-163

[B23] Hung R. Lee S. Bennett J. W. (2013). *Arabidopsis thaliana* as a model system for testing the effect of *Trichoderma* volatile organic compounds. Fungal Ecol. 6, 19–26. 10.1016/j.funeco.2012.09.005

[B24] Jefferson R. A. Kavanagh T. A. Bevan M. W. (1987). GUS fusions: beta-glucurodinase as a sensitive and versatile gene fusion marker in higher plants. EMBO J. 6, 3901–3907. 3327686 10.1002/j.1460-2075.1987.tb02730.x PMC553867

[B25] Kai M. Piechulla B. (2009). Plant growth promotion due to rhizobacterial volatiles - An effect of CO2? FEBS Lett. 583, 3473–3477. 10.1016/j.febslet.2009.09.053 19808036

[B26] Kang S. Demers J. del Mar Jimenez-Gasco M. Rep M. (2014). Fusarium oxysporum in Genomics of Plant-Associated Fungi and Oomycetes: Dicot Pathogens, eds Dean R. A. Lichens-Park A. Chittaranjan K. (Berlin, Heidelberg; Springer), 99–119.

[B27] Keller C. P. Stahlberg R. Barkawi L. S. Cohen J. D. (2004). Long-term inhibition by auxin of leaf blade expansion in bean and *Arabidopsis*. Plant Physiol. 134, 1217–1226. 10.1104/pp.103.032300 14988474 PMC389946

[B28] Kepinski S. Leyser O. (2005). The *Arabidopsis* F-box protein TIR1 is an auxin receptor. Nature 435, 446–451. 10.1038/nature03542 15917798

[B29] Kidd B. N. Kadoo N. Y. Dombrecht B. Tekeoğlu M. Gardiner D. M. Thatcher L. F.. (2011). Auxin signaling and transport promote susceptibility to the root-infecting fungal pathogen *Fusarium oxysporum* in *Arabidopsis*. Mol. Plant Microbe Interact. 24, 733–748. 10.1094/MPMI-08-10-0194 21281113

[B30] Kramer R. Abraham W.-R. (2012). Volatile sesquiterpenes from fungi: what are they good for? Phytochem. Rev. 11, 15–37. 10.1007/s11101-011-9216-2

[B31] Kwon Y. S. Ryu C. M. Lee S. Park H. B. Han K. S. Lee J. H.. (2010). Proteome analysis of *Arabidopsis* seedlings exposed to bacterial volatiles. Planta 232, 1355–1370. 10.1007/s00425-010-1259-x 20820802

[B32] Lucas M. Guédon Y. Jay-Allemand C. Godin C. Laplaze L. (2008). An auxin transport-based model of root branching in *Arabidopsis thaliana*. PLoS ONE 3:e3673. 10.1371/journal.pone.0003673 18989371 PMC2577305

[B33] Luschnig C. Gaxiola R. A. Grisafi P. Fink G. R. (1998). EIR1, a root-specific protein involved in auxin transport, is required for gravitropism in *Arabidopsis thaliana*. Genes Dev. 12, 2175–2187. 10.1101/gad.12.14.2175 9679062 PMC317016

[B34] Malcolm G. M. Kuldau G. A. Gugino B. K. Jiménez-Gasco M. M. (2013). Hidden host plant associations of soilborne fungal pathogens: an ecological perspective. Phytopathology 103, 538–544. 10.1094/PHYTO-08-12-0192-LE 23301815

[B35] Marchant A. Kargul J. May S. T. Muller P. Delbarre A. Perrot-Rechenmann C.. (1999). AUX1 regulates root gravitropism in *Arabidopsis* by facilitating auxin uptake within root apical tissues. EMBO J. 18, 2066–2073. 10.1093/emboj/18.8.2066 10205161 PMC1171291

[B36] Michielse C. B. Rep M. (2009). Pathogen profile update: *Fusarium oxysporum*. Mol. Plant Pathol. 10, 311–324. 10.1111/j.1364-3703.2009.00538.x 19400835 PMC6640313

[B37] Minerdi D. Bossi S. Maffei M. E. Gullino M. L. Garibaldi A. (2011). *Fusarium oxysporum* and its bacterial consortium promote lettuce growth and expansin A5 gene expression through microbial volatile organic compound (MVOC) emission. FEMS Microbiol. Ecol. 76, 342–351. 10.1111/j.1574-6941.2011.01051.x 21255049

[B38] Mitchum M. G. Yamaguchi S. Hanada A. Kuwahara A. Yoshioka Y. Kato T.. (2006). Distinct and overlapping roles of two gibberellin 3-oxidases in *Arabidopsis* development. Plant J. 45, 804–818. 10.1111/j.1365-313X.2005.02642.x 16460513

[B39] Morath S. U. Hung R. Bennett J. W. (2012). Fungal volatile organic compounds: a review with emphasis on their biotechnological potential. Fungal Biol. Rev. 26, 73–83. 10.1016/j.fbr.2012.07.001

[B40] Murashige T. Skoog F. (1962). A revised medium for rapid growth and bio assays with tobacco tissue cultures. Physiol. Plant. 15, 473–497. 10.1111/j.1399-3054.1962.tb08052.x

[B41] Naznin H. A. Kimura M. Miyazawa M. Hyakumachi M. (2013). Analysis of volatile organic compounds emitted by plant growth-promoting fungus *Phoma* sp GS8-3 for growth promotion effects on tobacco. Microbes Environ. 28, 42–49. 10.1264/jsme2.ME12085 23080408 PMC4070678

[B42] O'Donnell K. Gueidan C. Sink S. Johnston P. R. Crous P. W. Glenn A.. (2009). A two-locus DNA sequence database for typing plant and human pathogens within the *Fusarium oxysporum* species complex. Fungal Genet. Biol. 46, 936–948. 10.1016/j.fgb.2009.08.006 19715767

[B43] Okmen B. Doehlemann G. (2014). Inside plant: biotrophic strategies to modulate host immunity and metabolism. Curr. Opin. Plant Biol. 20, 19–25. 10.1016/j.pbi.2014.03.011 24780462

[B44] Ospina-Giraldo M. D. Mullins E. Kang S. (2003). Loss of function of the *Fusarium oxysporum* SNF1 gene reduces virulence on cabbage and *Arabidopsis*. Curr. Genet. 44, 49–57. 10.1007/s00294-003-0419-y 12845476

[B45] Péret B. De Rybel B. Casimiro I. Benková E. Swarup R. Laplaze L.. (2009). *Arabidopsis* lateral root development: an emerging story. Trends Plant Sci. 14, 399–408. 10.1016/j.tplants.2009.05.002 19559642

[B46] Poorter H. Navas M. L. (2003). Plant growth and competition at elevated CO2: on winners, losers and functional groups. New Phytol. 157, 175–198. 10.1046/j.1469-8137.2003.00680.x 33873640

[B47] Raaijmakers J. M. Paulitz T. C. Steinberg C. Alabouvette C. Moenne-Loccoz Y. (2009). The rhizosphere: a playground and battlefield for soilborne pathogens and beneficial microorganisms. Plant Soil 321, 341–361. 10.1007/s11104-008-9568-6

[B48] Rispail N. Di Pietro A. (2009). *Fusarium oxysporum* Ste12 controls invasive growth and virulence downstream of the Fmk1 MAPK cascade. Mol. Plant Microbe Interact. 22, 830–839. 10.1094/MPMI-22-7-0830 19522565

[B49] Rovenich H. Boshoven J. C. Thomma B. P. H. J. (2014). Filamentous pathogen effector functions: of pathogens, hosts and microbiomes. Curr. Opin. Plant Biol. 20, 96–103. 10.1016/j.pbi.2014.05.001 24879450

[B50] Ryu C. M. Farag M. A. Hu C.-H. Reddy M. S. Kloepper J. W. Paré P. W. (2004). Bacterial volatiles induce systemic resistance in *Arabidopsis*. Plant Physiol. 134, 1017–1026. 10.1104/pp.103.026583 14976231 PMC389924

[B51] Ryu C. M. Farag M. A. Hu C.-H. Reddy M. S. Wei H.-X. Paré P. W.. (2003). Bacterial volatiles promote growth in *Arabidopsis*. Proc. Natl. Acad. Sci. U.S.A. 100, 4927–4932. 10.1073/pnas.0730845100 12684534 PMC153657

[B52] Schaller G. E. Ladd A. N. Lanahan M. B. Spanbauer J. M. Bleecker A. B. (1995). The ethylene response mediator ETR1 from *Arabidopsis* forms a disulfide-linked dimer. J. Biol. Chem. 270, 12526–12530. 10.1074/jbc.270.21.12526 7759498

[B53] Smart R. E. Bingham G. E. (1974). Rapid estimates of relative water content. Plant Physiol. 53, 258–260. 10.1104/pp.53.2.258 16658686 PMC541374

[B54] Splivallo R. Novero M. Bertea C. M. Bossi S. Bonfante P. (2007). Truffle volatiles inhibit growth and induce an oxidative burst in *Arabidopsis thaliana*. New Phytol. 175, 417–424. 10.1111/j.1469-8137.2007.02141.x 17635217

[B55] Tsavkelova E. A. Klimova S. Y. Cherdyntseva T. A. Netrusov A. I. (2006). Microbial producers of plant growth stimulators and their practical use: a review. Appl. Biochem. Microbiol. 42, 117–126. 10.1134/S0003683806020013 16761564

[B56] Turlier M. F. Eparvier A. Alabouvette C. (1994). Early dynamic interactions between *Fusarium oxysporum* f.sp. lini and the roots of *Linum usitatissium* as revealed by transgenic GUS-marked hyphae. Can. J. Bot. 72, 1605–1612. 10.1139/b94-198

[B57] Vespermann A. Kai M. Piechulla B. (2007). Rhizobacterial volatiles affect the growth of fungi and *Arabidopsis thaliana*. Appl. Environ. Microbiol. 73, 5639–5641. 10.1128/AEM.01078-07 17601806 PMC2042089

[B58] Weiberg A. Wang M. Bellinger M. Jin H. (2014). Small RNAs: a new paradigm in plant-microbe interactions. Annu. Rev. Phytopathol. 52, 495–516. 10.1146/annurev-phyto-102313-045933 25090478

[B59] Yang X. Q. Lee S. So J. H. Dharmasiri S. Dharmasiri N. Ge L.. (2004). The IAA1 protein is encoded by AXR5 and is a substrate of SCF(TIR1). Plant J. 40, 772–782. 10.1111/j.1365-313X.2004.02254.x 15546359

[B60] Yang Y. D. Hammes U. Z. Taylor C. G. Schachtman D. P. Nielsen E. (2006). High-affinity auxin transport by the AUX1 influx carrier protein. Curr. Biol. 16, 1123–1127. 10.1016/j.cub.2006.04.029 16677815

[B61] Young G. (2009). Fungal pathogenesis: fungal communication gets volatile. Nat. Rev. Microbiol. 7, 6 10.1038/nrmicro2064

[B62] Zhang H. Kim M.-S. Krishnamachari V. Payton P. Sun Y. Grimson M.. (2007). Rhizobacterial volatile emissions regulate auxin homeostasis and cell expansion in *Arabidopsis*. Planta 226, 839–851. 10.1007/s00425-007-0530-2 17497164

