# Supplementary material for: Fusarium Oxysporum Volatiles Enhance Plant Growth Via Affecting Auxin Transport and Signaling
Source: Front Microbiol. 2015 Nov 10;6:1248. doi: 10.3389/fmicb.2015.01248 (PMC4639627; doi:10.3389/fmicb.2015.01248)
Supplement: Supplementary file 1 [file DataSheet1.DOCX]

**
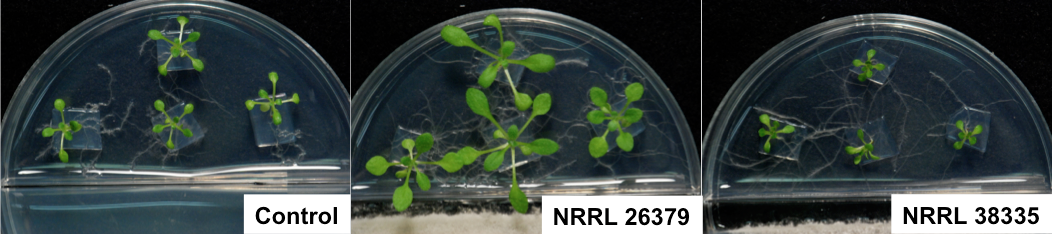
**Supplementary Fig. 1**.** Growth response of ecotype C24 to volatiles produced by two *F. oxysporum* isolates that enhanced Col-0 growth. Seedlings after two weeks of cocultivation with NRRL 26379 and NRRL 38335 are shown.

**
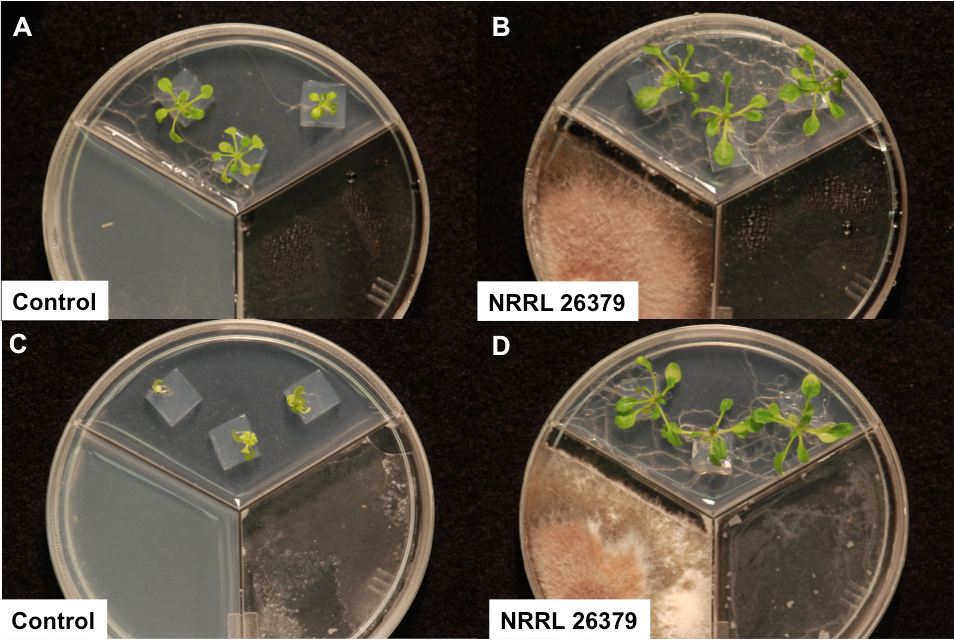
**Supplementary Fig. 2. Effect of CO_2_ on plant growth. Cocultivation of Col-0 with no fungus (control) and NRRL 26379, a growth enhancing isolate, in the absence (**A** and **B**) and presence (**C** and **D**) of Ba(OH)_2_ in Y plates.

**
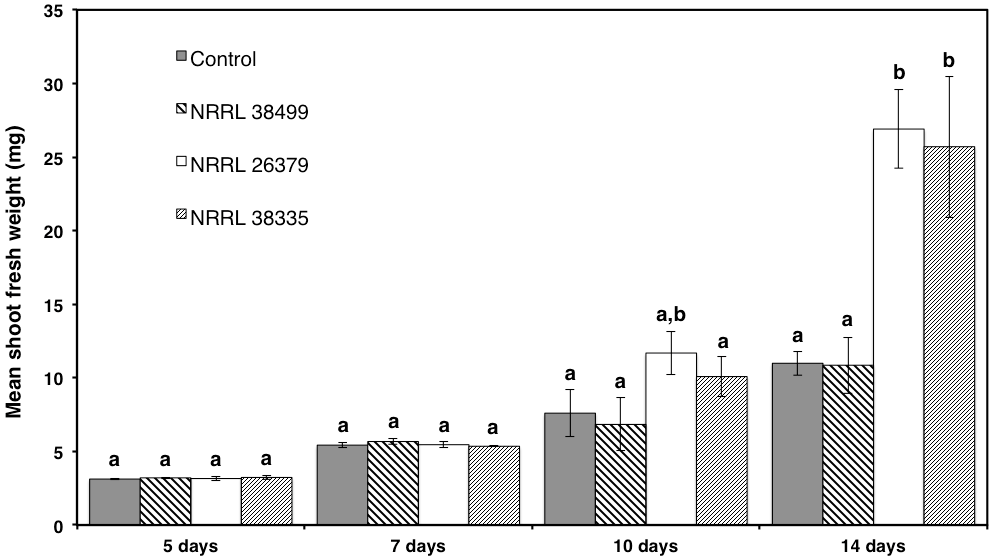
**

Supplementary Fig. 3**.** Progress of plant growth during cocultivation with *F. oxysporum*. The shoot fresh weight of Col-0 seedlings was measured after 5, 7, 10 and 14 days of cocultivation with no fungus (control), NRRL 38499, NRRL 26379 and NRRL 38335. Means and standard errors for two biological replicates per treatment, with five seedlings per replicate, are shown. Different letters on columns denote statistically significant differences within each time point by one-way analysis of variance.

**
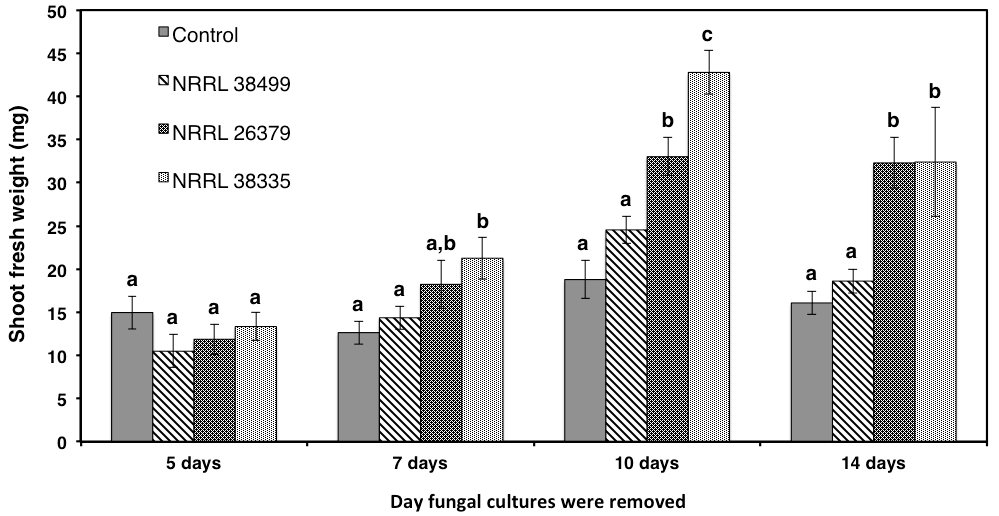
**

**
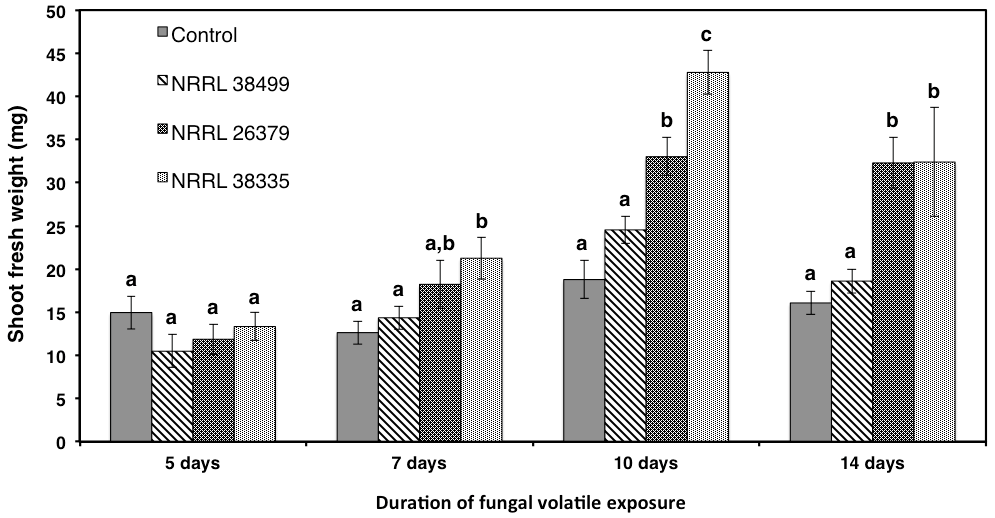
**

Supplementary Fig. 4**.** Correlation between the degree of growth enhancement and the duration of *F. oxysporum* volatile exposure. After setting up cocultivation with Col-0 seedlings, cultures of NRRL 38499, NRRL 26379 and NRRL 38335 were removed at 5, 7 and 10 days after inoculation. The resulting shoot fresh weight under all treatments was measured at day 14. Means and standard errors for three biological replicates per treatment, with five seedlings per replicate, are shown. Different letters on columns represent statistically significant differences by one-way analysis of variance.

**
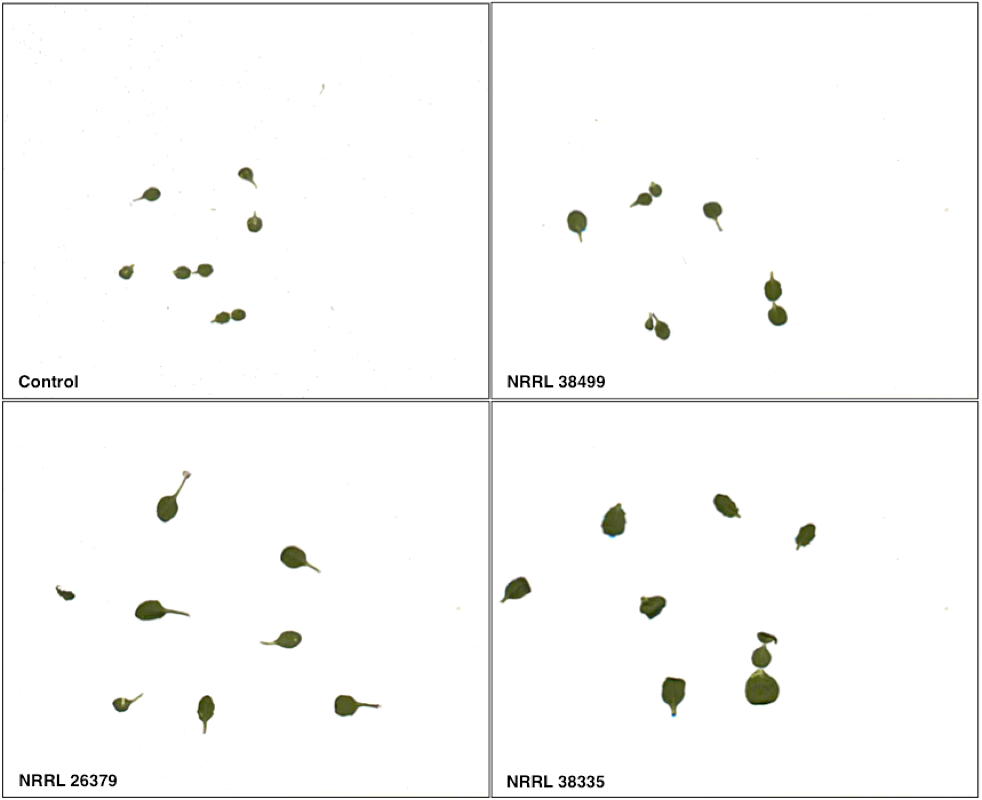
**

Supplementary Fig. 5. Leaf size after exposure to *F*. *oxysporum* volatiles. Representative leaves of Col-0 collected after cocultivation with no fungus (control), NRRL 38499, NRRL 26379 and NRRL 38335 for 14 days are shown.

**
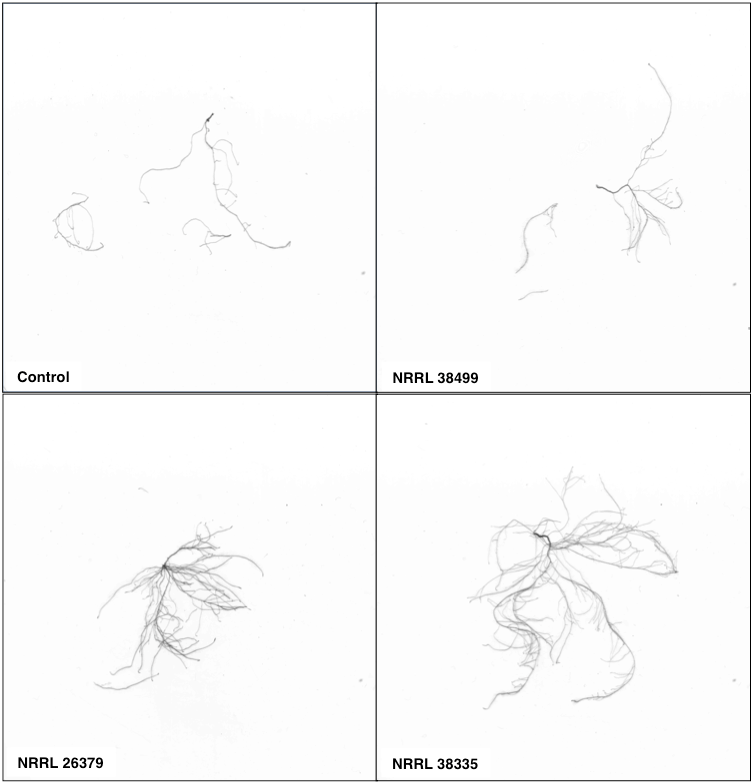
**

Supplementary Fig. 6. Roots of *A. thaliana* after exposure to *F*. *oxysporum* volatiles. Representative roots of Col-0 collected after cocultivation with no fungus (control), NRRL 38499, NRRL 26379 and NRRL 38335 for 14 days are shown.

**
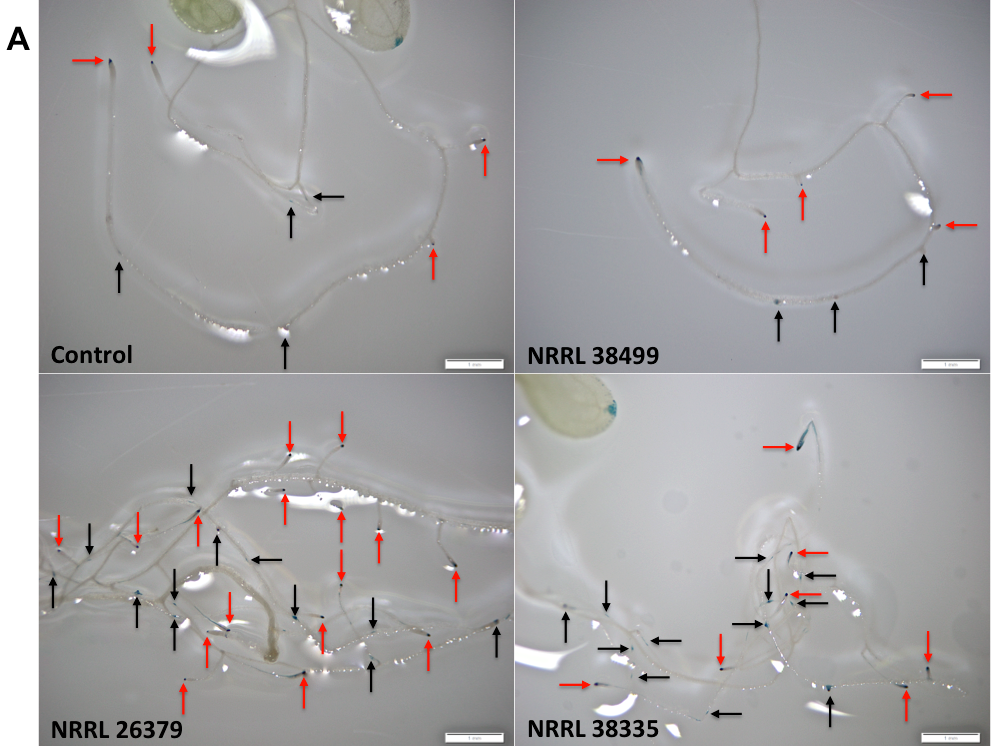
**

**
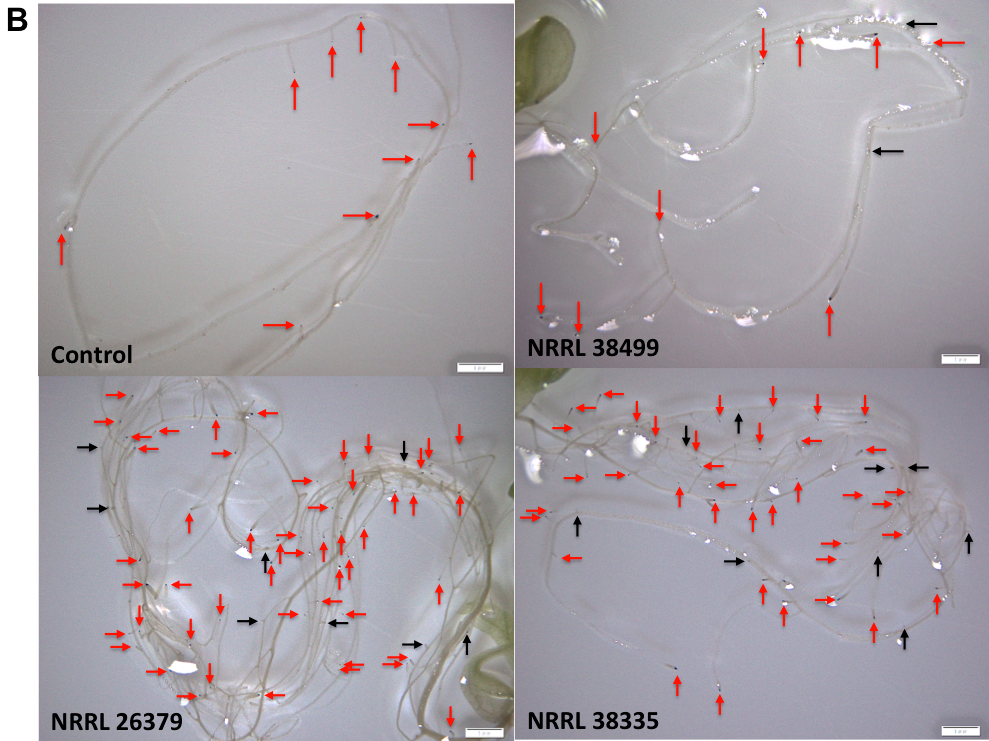
**Supplementary Fig. 7**.** GUS activity in roots of the Col-0 *DR5::GUS* line after cocultivation with *F. oxysporum*. After cocultivation with no fungus (control), NRRL 38499, NRRL 26379 and NRRL 38335, GUS staining was performed. Root tips and lateral root primordia expressing GUS after 7 (**A**) and 14 (**B**) days of cocultivation are marked with red and black arrows, respectively. Scale bar = 1mm.


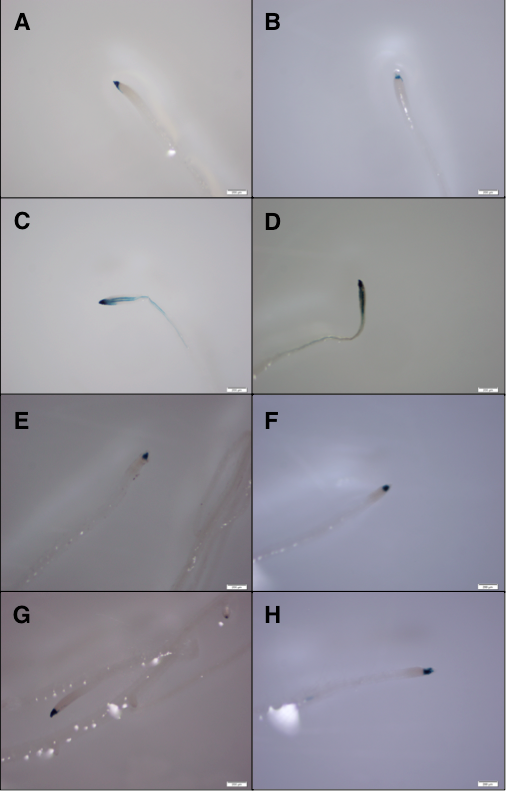


Supplementary Fig. 8**.** Enlarged view of GUS activity in root tips of the Col-0 *DR5::GUS* line after cocultivation with *F. oxysporum*. Selected root tips after 7 (**A**-**D**) and 14 (**E**-**F**) days of cocultivation with no fungus (**A** and **E**), NRRL 38499 (**B** and **F**), NRRL 26379 (**C** and **G**) and NRRL 38335 (**D** and **H**) are shown. Scale bar = 200μm.

**
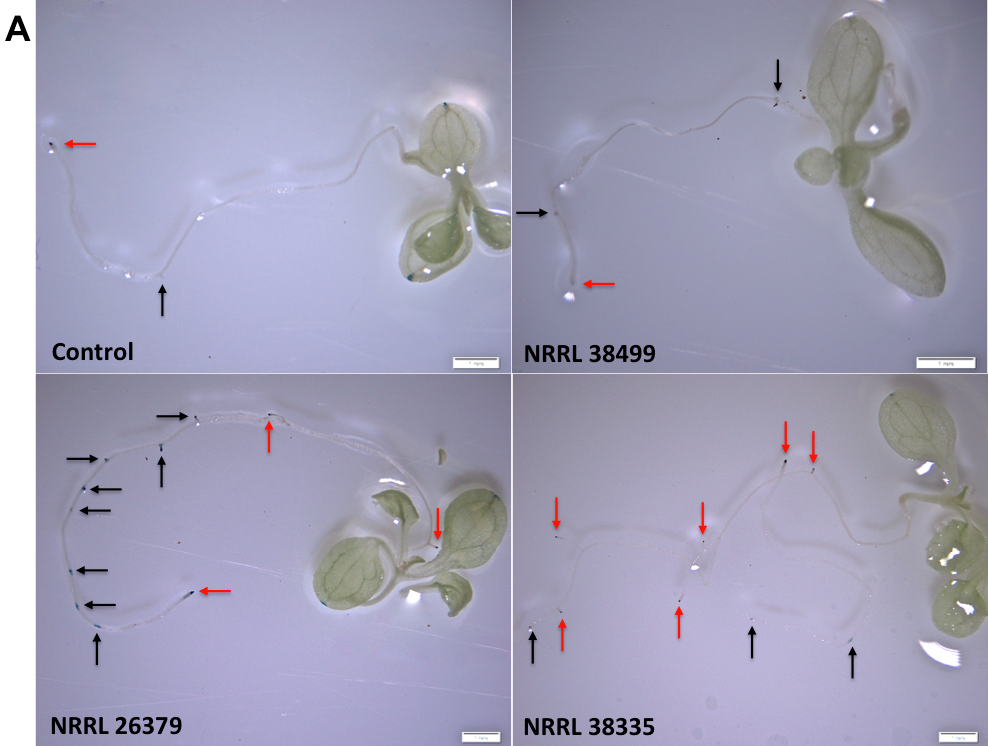
**

**
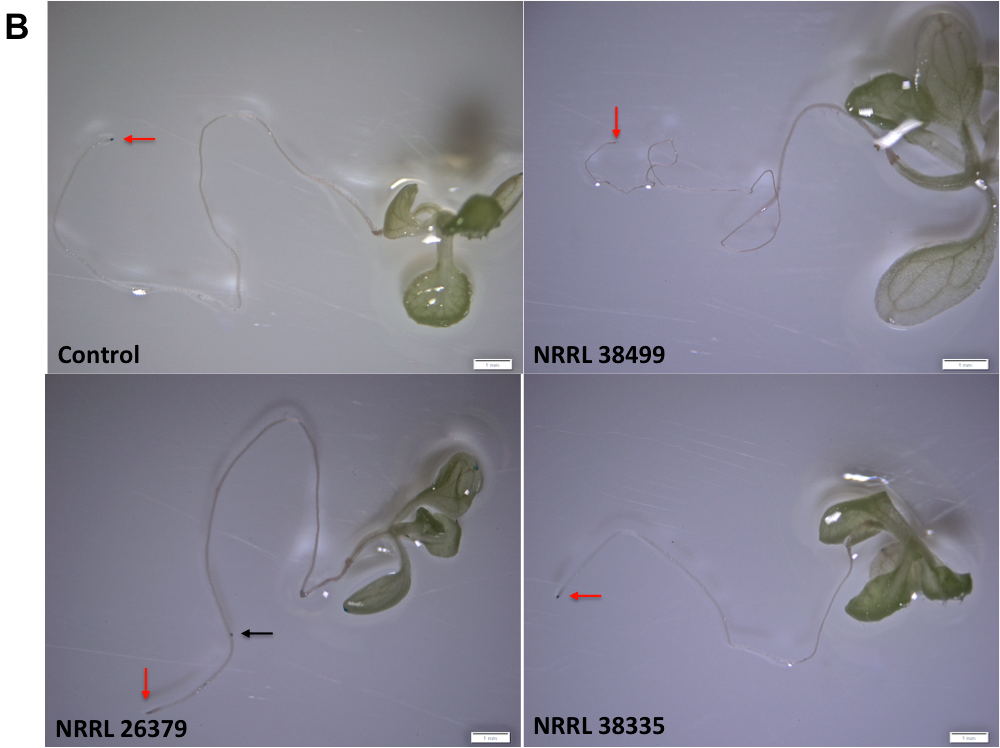
**


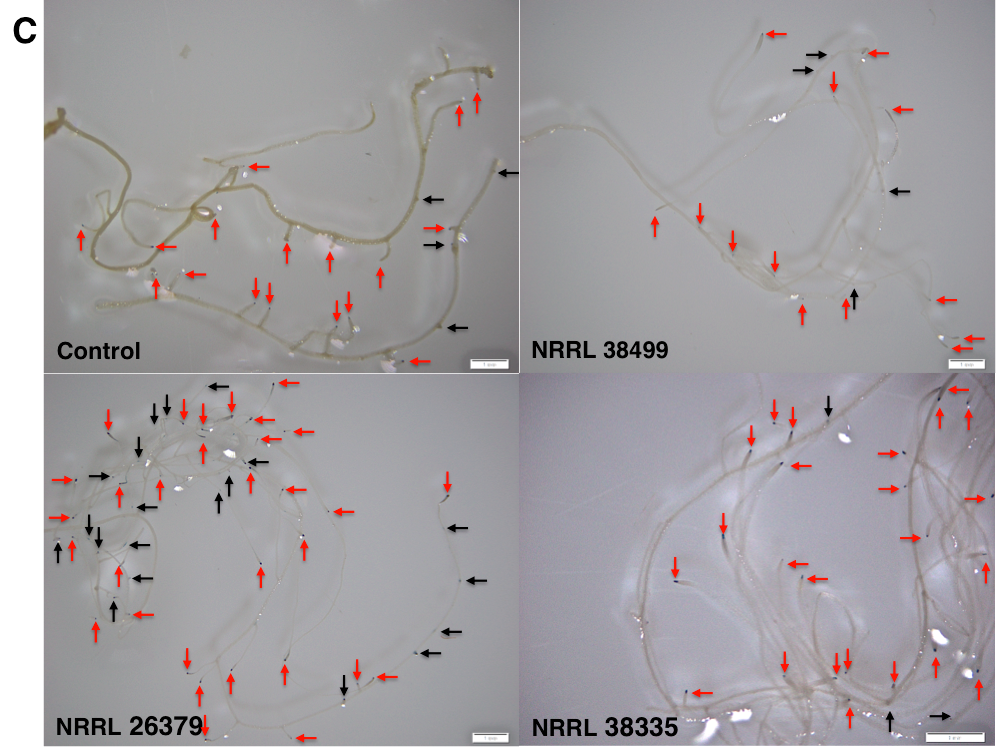
**
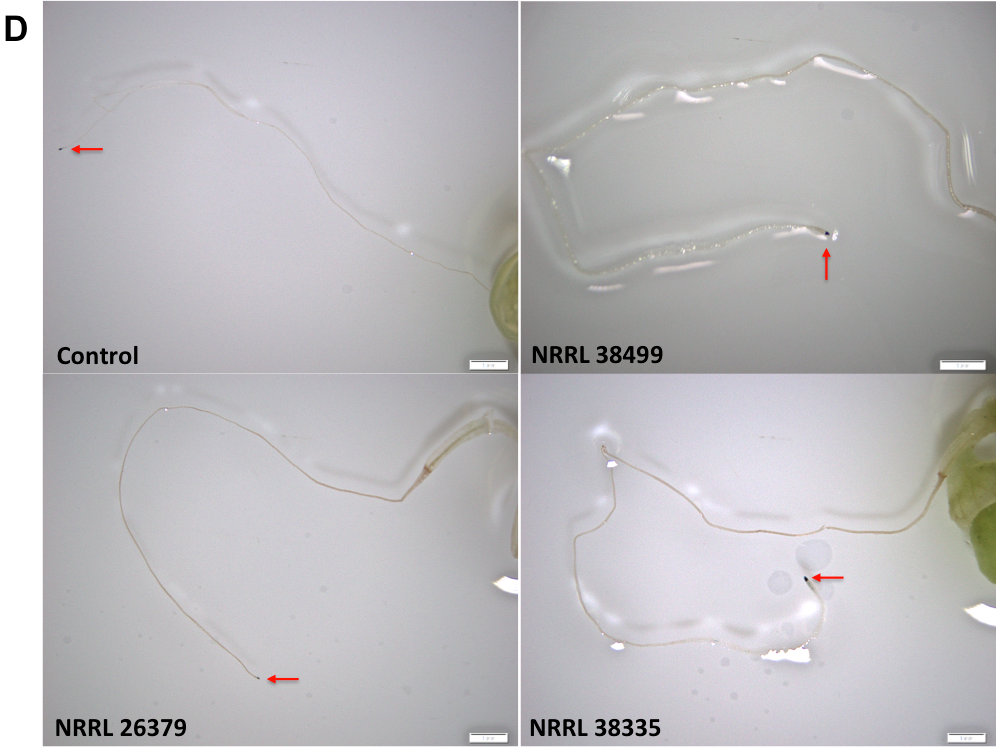
**Supplementary Fig. 9**.** Effect of NPA on volatile-mediated lateral root development. The Col-0 *DR5::GUS* line, grown on MS amended with 1μM NPA (**A** and **C**) and 5μM NPA (**B** and **D**), were cocultivated with no fungus (control), NRRL 38499, NRRL 26379 and NRRL 38335. Root tips and lateral root primordia expressing GUS after 7 (**A** and **B**) and 14 (**C** and **D**) days of cocultivation are marked with red and black arrows, respectively. Scale bar = 1 mm.

**
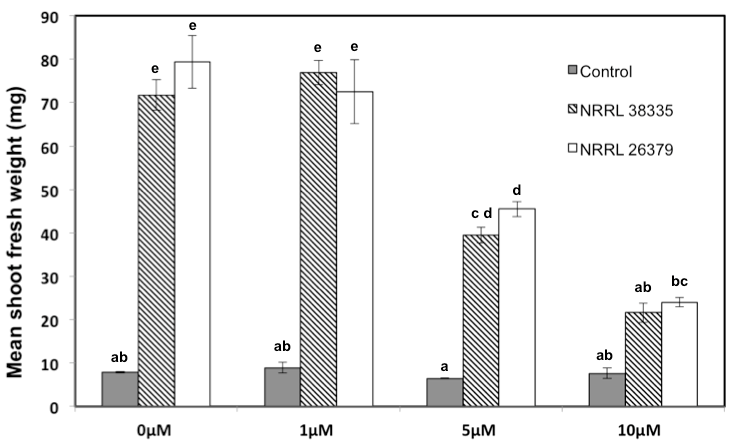
**

Supplementary Fig. 10**.** Effect of NPA on tobacco growth. The shoot fresh weight of tobacco seedlings grown on MS amended with 1μM, 5μM and 10μM NPA, was measured after cocultivation with no fungus (control), NRRL 38335 and NRRL 26379 for 14 days. Means and standard errors for three biological replicates per treatment, with five seedlings per replicate, are shown. Different letters on columns denote statistically significant differences by one-way analysis of variance.


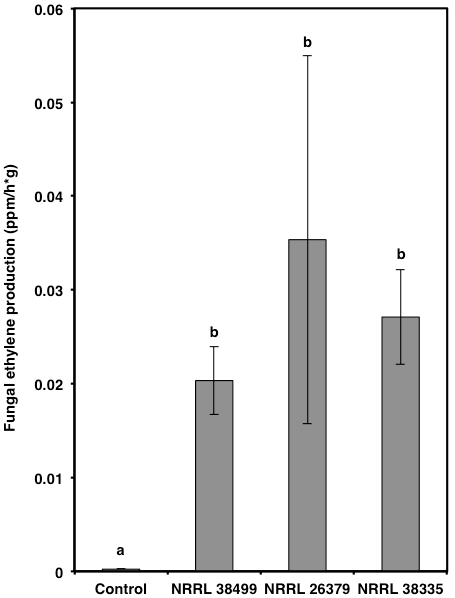


Supplementary Fig. 11**.** Ethylene production by *F. oxysporum*. Ethylene production rates from no fungus (control), NRRL 38499, NRRL 26379 and NRRL 38335 cultured on Potato Dextrose Broth are shown. Means and standard errors for three biological replicates per treatment are shown. Different letters on columns indicate statistically significant differences by one-way analysis of variance.
